# The relationship between transposable elements and ecological niches in the Greater Cape Floristic Region: A study on the genus *Pteronia* (Asteraceae)

**DOI:** 10.3389/fpls.2022.982852

**Published:** 2022-09-29

**Authors:** Zuzana Chumová, Alexander Belyayev, Terezie Mandáková, Vojtěch Zeisek, Eva Hodková, Kristýna Šemberová, Douglas Euston-Brown, Pavel Trávníček

**Affiliations:** ^1^Institute of Botany of the Czech Academy of Sciences, Průhonice, Czechia; ^2^Department of Botany, Faculty of Science, Charles University, Prague, Czechia; ^3^Central European Institute of Technology (CEITEC), Masaryk University, Brno, Czechia; ^4^Faculty of Science, Masaryk University, Brno, Czechia; ^5^Independent botanist, Scarborough, South Africa

**Keywords:** genome size, Greater Cape Floristic Region (GCFR), HybSeq, niche modelling, *Pteronia*, repeatome

## Abstract

Non-coding repetitive DNA (repeatome) is an active part of the nuclear genome, involved in its structure, evolution and function. It is dominated by transposable elements (TEs) and satellite DNA and is prone to the most rapid changes over time. The TEs activity presumably causes the global genome reorganization and may play an adaptive or regulatory role in response to environmental challenges. This assumption is applied here for the first time to plants from the Cape Floristic hotspot to determine whether changes in repetitive DNA are related to responses to a harsh, but extremely species-rich environment. The genus *Pteronia* (Asteraceae) serves as a suitable model group because it shows considerable variation in genome size at the diploid level and has high and nearly equal levels of endemism in the two main Cape biomes, Fynbos and Succulent Karoo. First, we constructed a phylogeny based on multiple low-copy genes that served as a phylogenetic framework for detecting quantitative and qualitative changes in the repeatome. Second, we performed a comparative analysis of the environments of two groups of *Pteronia* differing in their TEs bursts. Our results suggest that the environmental transition from the Succulent Karoo to the Fynbos is accompanied by TEs burst, which is likely also driving phylogenetic divergence. We thus hypothesize that analysis of rapidly evolving repeatome could serve as an important proxy for determining the molecular basis of lineage divergence in rapidly radiating groups.

## Introduction

The time-space variability of the eukaryotic genome under the influence of climate change has been one of the key issues in biology for more than a hundred years although still not fully understood. Genome evolution is a heterogeneous process, and different genomic fractions change at different rates. The fraction of the nuclear genome that changes most rapidly over time and most likely responds to changing abiotic factors is non-coding repetitive DNA (= repeatome; McClintock, 1984; Wessler, 1996; Belyayev, 2014; Balao et al., 2017). The repeatome consists of several classes, predominated by transposable elements (TEs) and satellite DNA (satDNA) (Biscotti et al., 2015; Wei et al., 2018). Among the internal sources for genotypic change, TEs can be considered the most powerful due to their ability to move, to insert at novel locations and thereby to shape and specialize the landscapes of coding and non-coding DNA fractions of the eukaryotic genome. The process of genome change can be described as TE-driven genomic expansion on genome architecture with successive selection for specific gene arrangements and genome streamlining in successful lineages (Koonin, 2009). It is clear that genome remodelling driven by mobilized TEs enables escape from stasis and generates the genetic innovations needed for rapid diversification (Oliver and Greene, 2009; Zeh et al., 2009; Belyayev, 2014). The TEs, particularly retrotransposons, can be activated by biotic and abiotic stresses (Wessler, 1996; Balao et al., 2017). Barbara McClintock furthermore referred to “genome shock” as an activator of TEs (1984).

Another big repeatome component, satDNA, consists of long, late-replicating, non-coding arrays of tandemly arranged monomers (Biscotti et al., 2015; Satović et al., 2016; Camacho et al., 2022). These sequences are often species or genus specific and are considered the most variable fraction of the eukaryotic genome, thus reflecting trajectories of short-term evolutionary change (Charlesworth et al., 1994; Raskina et al., 2008). Recent studies suggest that satDNA, predominantly concentrated in the heterochromatic regions of chromosomes, is involved in various functions ranging from chromosome organization and pairing to cell metabolism and adjustment of gene functions (Martienssen, 2003; Mehrotra and Goyal, 2014; Garrido-Ramos, 2015; Meštrović et al., 2015). Given the properties of the major repeatome fractions, analysis of their changes can provide clues to the problem of ecology-dependent eukaryotic genome transformations.

One way to define the role of any genomic component is to project its dynamics into the evolution of a known biological system. Therefore, the aim of this study is to analyze next-generation sequencing (NGS) data on repeatome variation in the phylogenetic context of species in the genus *Pteronia*.

*Pteronia* L. (Asteraceae) is a southern African endemic genus comprising about 70 species (Hutchinson and Phillips, 1917; Kolberg and van Slageren, 2014; Bello, 2018), with a centre of diversity in the Greater Cape Floristic Region (GCFR). It is an evergreen, woody perennial genus that often contains aromatic substances. *Pteronia* is considered to be isolated among the African Compositae in a separate subtribe Pteroniinae Nesom. (Nesom, 2020) and most closely related to Homochrominae Benth. (comprising e.g. *Felicia* Cass., *Amellus* L., *Engleria* O. Hoffm. or *Chrysocoma* L.). *Pteronia* species can mostly be found in dry habitats, particularly in the Succulent Karoo and to a lesser extent in the Nama Karoo biomes (Manning and Goldblatt, 2012; Snijman, 2013). However, some species are exclusively tied to Fynbos.

Fynbos and Succulent Karoo biomes make up the majority of the Greater Cape Floristic Region (Bergh et al., 2014), which is climatically characterized by winter rainfall and summer drought. It is the smallest of the world’s six recognised floristic kingdoms (the core part of it was recognised by Good (1947)), with exceptionally high diversity and endemism; more than 11 000 species of vascular plants are found here, more than 70% of which are endemic (72% estimated by Born et al. (2007) or 78% by Snijman (2013)). The Succulent Karoo is associated with drier and warmer conditions, and is found on plains and lower slopes with annual rainfall between 20 and 300 mm and summer temperatures reaching up to 44 °C, while Fynbos is found on sandy lowland coastal plains as well as mountains, but not in areas where annual rainfall is below 200 mm, and less commonly on shale-derived loamy mesotrophic substrates (Low et al., 1996; Mucina et al., 2006; Rebelo et al., 2006).

In addition to climate, geology and soil, disturbance is considered to be one of the main factors shaping vegetation in southern Africa (Bond, 1997; Bond et al., 2003). The Fynbos biome is adapted to fires, which drive ecological processes such as regeneration, succession and vegetation dynamics (Bond and van Wilgen, 1996; Keeley et al., 2012). Conversely, Succulent Karoo species are intolerant of fire but in the absence of fire, some typical representatives such as thicket and succulents have successfully invaded the Fynbos (Cowling and Pierce, 1988). Some species of the genus *Pteronia* thus must be able to cope with these different conditions as they inhabit both biomes, while other species are restricted to one biome and need only cope with its particular conditions.

Given the linkage of some species of the genus *Pteronia* to different ecological conditions in a unique South African botanical region and the considerable differences in genome size at the likely diploid level, we hypothesized the possibility of different dynamics of repeatome components in different lineages with different ecological requirements. Therefore, the aim of this study is to investigate the quantitative and qualitative parameters of TE and major satDNA families for different diploid lineages and to perform a comparative analysis in relation to their ecology. Specifically, this involves reconstructing phylogeny using the method of target enrichment of low-copy nuclear genes, assessing the evolution of genome size and its relationship to the accumulation of repetitive DNA in the phylogenetic context, estimating groups of TEs and other repetitive elements that have a major influence on genome composition, and identifying possible links to environmental factors associated with their proliferation. At the molecular level, these data may shed light on speciation patterns and vectors of species divergence under variable ecological conditions.

## Material and methods

### Taxon sampling

**Figure 1:**
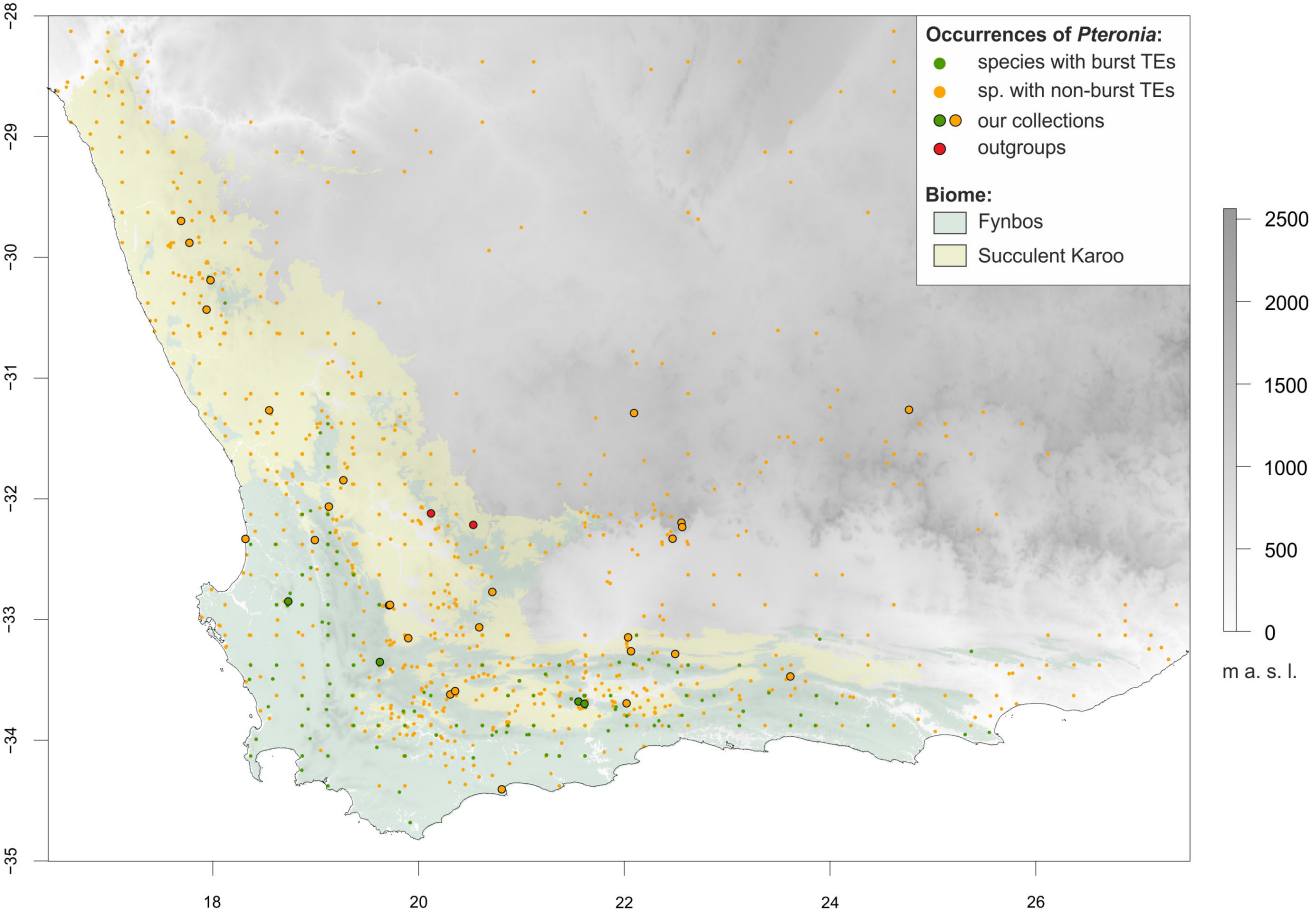
Topographic map of southern Africa highlighting the two dominant biomes of the Greater Cape Floristic Region, Fynbos (light green) and Succulent Karoo (yellow), and the distribution of the *Pteronia* species studied. Occurrence points are colour-coded according to transposable element (TEs) multiplication **–** dark green for species with burst TEs, orange for species with non-burst TEs (see text for details). Sampling points are circled in black. The extent of the map is adjusted for environmental niche modelling, where the full extent corresponds to the area with topographic data availability, whereas the shaded area (biomes) corresponds to the area with soil data availability.

For this study, 32 populations of the genus *Pteronia* (114 individuals) and two other species subsequently used as outgroups (*Chrysocoma ciliata* L. and *Oedera glandulosa* (Thunb.) N.G.Bergh) were selected from our 2018–2019 collections in the Greater Cape Floristic Region (**Figure 1**, **Supplementary Table 1**). Several selection criteria were used in order to reduce the species richness of the genus to a manageable level, but at the same time to maximize the variability of the selected representatives of the genus in terms of morphological and environmental variability. In the first step, diploid species were selected based on an extensive screening of genome sizes across the genus’ range. In the next step, only plant material that was clearly identifiable and taxonomically classifiable was selected. The final step of selection was ecological distinctiveness, where species that showed large differences in ecological requirements according to their distribution were selected. The selection includes 31 diploid taxa of the genus *Pteronia*, one of which has two populations differing in genome size. At each locality, the following material was collected for each taxon, where possible: (1) flowering shoots (1–6 individuals, depending on population size) with well-developed intact leaves for flow-cytometry estimation of nuclear genome (fresh) and for molecular analyses (silica-dried); (2) mature achenes from several individuals for subsequent germination and cultivation in the experimental garden of the Institute of Botany of the Czech Academy of Sciences in Průhonice for karyology and genomic analyses; (3) herbarium vouchers (deposited in the PRA). While all collected samples were subjected to genome size estimation, the molecular analyses were performed on one selected specimen per population.

### DNA extraction

Total genomic DNA was extracted from 0.5 g of silica-dried leaf material by the Sorbitol method (Štorchová et al., 2000). The quality of the DNA was checked on 1% agarose gels and using a Qubit 2.0 fluorometer (Invitrogen, Carlsbad, CA, USA). This approach was used for one specimen of 32 *Pteronia* populations and for one specimen of *Oedera glandulosa* and three specimens of *Chrysocoma ciliata*.

### HybSeq library preparation

Approximately 800 ng of extracted DNA was sheared in 50 μl double-distilled H_2_O using an M220 Focused-ultrasonicator (Covaris, Woburn, MA, USA; settings 52 s, 6 °C, 200 cycles), ~500 bp fragment length was verified using agarose electrophoresis. Library preparation followed the NEBNext Ultra DNA Library Prep Kit for the Illumina protocol (New England Biolabs, Ipswich, MA, USA) with the following modifications. Half volumes of samples and NEBNext chemicals were used, an additional cleanup step after the adapter ligation was done using a QIAquick Purification Kit (QIAGEN, Venlo, Netherlands), and size selection to approximately 400–600 bp was performed using Agencourt AMPure XP beads (Beckman Coulter, Danvers, Massachusetts, USA). Amplification of the ligated, size-selected fragments was done using NEBNext Multiplex Oligos for Illumina Dual Index Primers Set 1 (New England Biolabs) and KAPA HiFi HotStart ReadyMix PCR Kit (Kappa Bioscience, Oslo, Norway). Purification of enriched PCR products was done twice using Agencourt AMPure XP beads at a 0.75 volume ratio.

The solution hybridization was done using the myBaits Expert Compositae-1061 target capture kit (Arbor Biosciences, Ann Arbor, Michigan, USA). The enrichment followed the myBaits manual v.4.01 with 800 ng DNA in 7 μl for a set of 24 samples and 12 cycles of PCR enrichment. Concentration of DNA was measured using a Qubit 2.0 fluorometer. Target-enriched libraries were mixed with unenriched libraries at a 1:1 ratio to increase the proportion of off-target cpDNA. The samples were sequenced on an Illumina (San Diego, California, USA) NovaSeq 6000 at IAB (Olomouc, Czech Republic) and/or on an Illumina MiSeq instrument at OMICS Genomika (Biocev, Vestec, Czech Republic), in both cases 150 bp paired-end reads were obtained.

### Processing of raw reads for HybSeq and species tree reconstruction from target enriched data

We used our samples of 32 species of *Pteronia*, three samples of *Chrysocoma ciliata* and one sample of *Oedera glandulosa* (**Supplementary Table 1**). We also added two samples downloaded from EMBL-EBI, which were sequenced with the same set of COS probes, *Chiliotrichum diffusum* SAMN11585365 and *Denekia capensis* SAMN11585369. All samples except *Pteronia* were later used as outgroups for the final species tree.

Pipeline to process our data is available at https://github.com/V-Z/hybseq-scripts. Raw FASTQ reads were trimmed by Trimmomatic 0.39 (Bolger et al., 2014) with settings “ILLUMINACLIP:adaptors.fa:2:30:10 SLIDINGWINDOW:5:20 LEADING:20 TRAILING:20 MINLEN:50” and deduplicated using clumpify.sh from bbamp2 38.42 (Bushnell et al., 2017). Quality of FASTQ files was checked by FastQC 0.11.5 (https://www.bioinformatics.babraham.ac.uk/projects/fastqc/). Contigs were recovered using HybPiper 1.3 (Johnson et al., 2016) with help of our wrapper scripts (https://github.com/V-Z/hybseq-scripts). HybPiper pipeline uses BBMap (https://sourceforge.net/projects/bbmap/), Biopython (Chapman & Chang, 2000; Cock et al., 2009), BLAST (Altschul et al., 1990; Altschul et al., 1997), BWA (Li, 2013), Exonerate (Slater & Birney, 2005), GNU Parallel (Tange, 2018), Samtools (Bonfield et al., 2021; Danecek et al., 2021) and SPAdes (Prjibelski et al., 2020). Contigs where less than ⅔ of all samples were recovered, were discarded. Passing contigs (exons recovered by HybPiper) were aligned by MAFFT 7.487 (Yamada et al., 2016) using default settings. Alignments were automatically trimmed by the R script hybseq_4_alignment_3_run.r (R 4.0.0, R Core Team, 2021) and library *ape* (Paradis and Schliep, 2019): all samples with more than 20% of missing data and then all alignment positions with more than 20% of missing data were removed. Alignment statistics were extracted from the output of ape::checkAlignment function. Alignments shorter than 200 bp, with less than ⅔ of samples or with more than 40% sites with 2 or more different bases (putative paralogs), were removed. We obtained branch supports with the 10 000 ultrafast bootstraps (Hoang et al., 2018); bootstrapped trees were optimized by NNI on bootstrap alignment) and 10 000 replicates for SH approximate likelihood ratio test implemented in the IQ-TREE 2.1.3 (Nguyen et al., 2015), which was used to reconstruct unrooted ML trees from passing alignments. The IQ-TREE started for each alignment with 99 parsimony and BIONJ starting tree (IQ-TREE default) and performed 5 runs with extended model selection followed by tree inference (taking into account invariable sites plus FreeRate model with up to 100 categories). Topologies of resulting gene trees were compared with ape::dist.topo (Kuhner and Felsenstein, 1994) and PCoA (ade4::dudi.pco, (Gower, 1966; Dray and Dufour, 2007). Outlying trees were removed. Trees with outlying topologies were further filtered out using *kdetrees* (dissimilarity distance matrix, IQR multiplier for outlier detection was 0.4) R package (Weyenberg et al., 2014). Abnormally long branches were removed from remaining trees by TreeShrink (Mai and Mirarab, 2018). Species trees were reconstructed by ASTRAL III (Sayyari and Mirarab, 2016; Zhang et al., 2018).

### Flow cytometry

Data on genome size and GC content were estimated *via* flow cytometric analyses in accordance with the best practice recommendations (Sliwinska et al., 2022) and according to the methodology described in detail by Doležel et al. (2007). The results of all included taxa were processed using FloMax v2.4d (Partec GmbH) and are given in **Supplementary Table 1**. Both types of analysis (one using the AT-selective fluorescent dye DAPI and the other the intercalating fluorochrome propidium iodide) were conducted with the same standard, which did not overlap with any signal of the sample. *Solanum pseudocapsicum* (1C = 1.295 pg, Temsch et al., 2010); GC content 38.52%) served as the primary standard, alternatively, *Pisum sativum* cv. ‘Ctirad’ (1C = 4.360 pg; GC content 41.77%) was used. The genome size of the secondary standard was estimated based on recalculation against the primary standard in simultaneous analyses that were repeated at least ten times on different days. GC content of both standards was adopted from Veselý et al. (2012), and the methodology of genome size as well as GC content estimation was adopted from Trávníček et al. (2019).

### Chromosome preparation

Actively growing young roots were collected from cultivated plants. The root tips were pre-treated with ice-cold water for 12 h, fixed in freshly prepared fixative (ethanol : acetic acid, 3 : 1) for 24 h at 4 °C and stored at −20 °C until further use. Chromosome spreads were prepared for 26 individuals (**Supplementary Table 1**) as described by Mandáková and Lysak (2016a). Chromosome preparations for fluorescent *in situ* hybridization (FISH; see below) were treated with 100 μg/ml RNase in 2× sodium saline citrate (SSC; 20× SSC : 3 M sodium chloride, 300 mM trisodium citrate, pH 7.0) for 60 min and with 0.1 mg/ml pepsin in 0.01 M HCl at 37 °C for 5 min; then postfixed in 4% formaldehyde in 2× SSC for 10 min, washed in 2× SSC twice for 5 min, and dehydrated in an ethanol series (70%, 90%, and 100%, 2 min each).

### Low-coverage whole-genome sequencing for repeatome analysis and plastome phylogeny

DNA sequencing libraries were prepared from all 32 *Pteronia* accessions and sequenced at the Admera Health Biopharma Services (New Jersey, USA) targeted for 1× coverage. The required DNA isolate aliquots (from the same samples used for the HybSeq libraries, see above) were sent to the company, the Kapa Hyper Prep approach was used to prepare the libraries, and the Illumina HiSeq X platform was used for sequencing, providing 150-bp for 49M paired-end reads per sample (24.5M in each direction). Raw FASTQ reads were pre-processed in the same way as HybSeq reads.

### Analyses of the partial chloroplast genome from low-coverage WGS

Complete chloroplast genome of *Erigeron breviscapus* (Vaniot) Hand.-Mazz. (Asteraceae, tribe Astereae; Yu et al., 2021; GenBank accession MN449489) was used as the reference sequence for the read mapping with intention to reconstruct the chloroplast genomes of *Pteronia* species *via* Bowtie 2 programme (Langmead and Salzberg, 2012) implemented in Geneious Prime software (v.2022.0.1; www.geneious.com; Kearse et al., 2012). The reads from low-coverage genome sequencing were used. The consensus sequences, with minimal coverage set to 10, were aligned *via* MAFFT together with the selection of 6 outgroups from tribes Astereae and Anthemideae that were available in GenBank (Astereaea – *Erigeron breviscapus* MN449489.1, *Aster ageratoides* Turcz. MW813970.1; Anthemideae – *Crossostephium chinense* (L.) Makino NC_042725.1, *Opisthopappus taihangensis* (Ling) C.Shih NC_042787.1, *Artemisia gmelinii* Weber ex Stechm. KU736962.1, *Artemisia lactiflora* Wall. ex DC. MW411453.1). Subsequently, the alignment positions with missing data for more than 10% of specimens were removed. The final alignment was analyzed *via* the ML approach implemented in Mega 11 software (Tamura et al., 2021) under GTR+G+I model, which was selected based on BIC comparison in jModelTest2 program (Darriba et al., 2012). Bootstrap supports were calculated based on 500 replicates. The final tree was time calibrated *via* the RelTime-ML approach in Mega 11 with a calibration point for divergence of the Astereae tribe and *Pteronia* genus adopted from Mandel et al. (2019). The time of node divergence was set to 11.96 Mya.

### Determination of repeatome composition

For processing the low-coverage WGS Illumina data and comparing the repetitive DNA fractions of the studied species, a public web server running RepeatExplorer2 (RE) (https://repeatexplorer-elixir.cerit-sc.cz/galaxy) was used (Novák et al., 2013). The discovery and characterization of repeatome elements of the particular genome was carried out by means of the “RepeatExplorer2 clustering” tool with default parameters. The information about the similarity hits was used to construct a graph where nodes represent sequence reads and edges between nodes correspond to similarity hits. Subsequently, the sequences were divided into the clusters based on the amount of similarity hits. Each cluster with a size above the default threshold was characterized by a similarity search against the databases of known repeats. The graphical layout of the clusters was calculated, resulting in an annotated genome composition that can be compared between tested species. After analyzing each species separately, a comparative analysis of the entire data set was performed (**Supplementary Figure 1**).

### Data processing and principal component analysis for TEs

For the estimation of the overall percentage of DNA sequences related to the certain TE in the genome, the annotated RE clusters were manually corrected. For genomically abundant clusters (> 0.01% of the genome), the percentages of quantitative genomic content were summarized (**Supplementary Table 2**) and the affiliation of a particular cluster to a supercluster was considered because some clusters were unclear in composition. Mitochondrion and plastome sequences were excluded from the analysis. PCA analysis using *prcomp* function in R was applied to evaluate level of divergence, to reduce the complexity and to retain most of the variation present in all of the original variables (Jolliffe, 2002). PCA was applied for the entire TEs and two dimensional PCA space was generated.

### Satellite DNA families determination and verification

The major satDNA families of *Pteronia* genomes were determined by tandem repeat analyzer TAREAN (Novák et al., 2017) within the framework of RE. The consensus monomers were checked for similarities with BLAST and for newly discovered satDNA families that did not show any similarities with the database, a conserved motif of 12 bp was determined for further *in silico* genome scanning (**Supplementary Table 3**, Belyayev et al., 2020). To verify presence or absence of tandem arrays within a certain genome, raw reads were assembled to contigs using Geneious Prime (GP) software. *De novo* assembly was performed with medium-low sensitivity, which is the best option for large numbers (e.g. 100 000 or more) of Illumina sequencing reads. The resulting contigs of the investigated species were scanned with the determined conserved motifs to identify the presence of the arrays of newly discovered satDNA families. Scanning was performed with the “search for motifs” command of the GP program, with a maximum of zero nucleotide mismatches. Contigs containing arrays of discovered satDNA families were further analyzed using the following two publicly available online tools: tandem repeat finder (TRF; https://tandem.bu.edu/trf/trf.html; Benson, 1999) and the YASS genomic similarity tool (http://bioinfo.lifl.fr/yass/yass.php; Noé and Kucherov, 2005), which enabled conformation of tandem organization.

### DNA probes

Synthetic oligonucleotide probes for *de novo* identified 168 bp, 83 bp, 194 bp and 112 bp tandem repeat “Family 1”, “Family 2”, “Family 3” and “Family 4”, respectively, were newly designed. Following target sequences (60 nt, with 45 and 55% GC content, respectively) were selected from DNA alignments using the Geneious v11.1.5 package to minimize self-annealing and formation of hairpin structures (**Supplementary Table 3**). All DNA probes were labeled with biotin-dUTP or digoxigenin-dUTP by nick translation as described previously (Mandáková and Lysak, 2016b).

### Fluorescence *in situ* hybridization, microscopy, and image processing

The labeled DNA probes were pooled together, ethanol precipitated, dissolved in a 20-μl mixture containing 50% formamide, 10% dextran sulfate and 2× SSC, and pipetted onto a pretreated and postfixed chromosome preparation. The slides (accessions marked in the **Supplementary Table 1**) were heated at 80 °C for 2 min and incubated at 37 °C overnight. Hybridized probes were visualized through fluorescently-labeled antibodies against biotin-dUTP (red) and digoxigenin-dUTP (green) as detailed in Mandáková and Lysak (2016b). Chromosomes were counterstained with 4’,6-diamidino-2-phenylindole (DAPI, 2 μg/ml) in Vectashield antifade. Fluorescence signals were analyzed and photographed using a Zeiss Axioimager epifluorescence microscope and a CoolCube camera (MetaSystems). Individual images were merged and processed using Photoshop CS (Adobe Systems).

### Whole-genome trait evolution

All data analyses were conducted in the R programming language v. 4.1.2 (R Core Team, 2021) with the use of packages allowing data analysis in the context of phylogeny. Ancestral states for genome size were reconstructed using the ‘fastAnc’ function (fast ML estimation under Brownian motion model; BM) and visualized on the tree with the ‘contMap’ function, both from the R package *phytools* v. 0.7-47 (Revell, 2012). To better understand the genome size evolution we applied several evolutionary models using the ‘transformPhylo.ML’ function from R package *motmot* (Thomas and Freckleton, 2012). All models were compared against the BM model (by calculation of AICc differentiation and by estimation of *p*-value using chi-squared distribution *via* ‘pchisq’ function) to evaluate the best fit. The test of the correlation between the whole genome size and genome size attributed to repeatome content was conducted in two ways – without phylogeny constraint and with phylogenetically independent contrast calculated in R package *ape* (Paradis and Schliep, 2019).

### Environmental niche modelling

Available environmental data related to possible eco-physiological constraints of the studied species were downloaded from various open-source databases. The first batch of data was annual trends and conditions related to precipitation and temperature (Bioclim variables) that were extracted from CHELSA v.2.1 (Karger et al., 2017). The second batch of data aimed specifically to the South Africa region was adopted from Wüest et al. (2019). This dataset contains additional climatic aggregations derived from CHELSA data on monthly basis, as well as topographic variables based on the digital elevation model GMTED2010 (Danielson and Gesch, 2011; data available at Dryad repository https://doi.org/10.5061/dryad.1cs77qn). The third data batch, for the GCFR only, contains soil data adopted from Cramer et al. (2019); data available at Dryad repository https://doi.org/10.5061/dryad.37qc017). All variables were downloaded at a resolution of 30 arcsec (~1 km).

Because of reduced availability of soil data, the data were analyzed in two extents – for the Cape provinces (Northern, Western and Eastern Cape), where all CHELSA and topographic data were extracted and used (corresponding to the map extent in **Figure 1**); and for the GCFR (corresponding to the shaded area in **Figure 1**), where all data (including soil) were extracted and used.

All downloaded environmental variables were examined for pairwise correlations (via vifcor function in *usdm* R-package, Naimi et al., 2014) based on extracted values for all georeferenced locations of *Pteronia* species. After exclusion of highly correlated variables (Pearson’s correlations > 0.70), 18 and 25 variables for both extents, respectively, were retained and used in further analyses (**Supplementary Table 4**).

Data on *Pteronia* occurrences are relatively scarce and besides our own field observations we used two database sources – Global Biodiversity Information Facility (GBIF; www.gbif.org) and iNaturalist (www.inaturalist.org). The data from databases were inspected for correct determination based on available picture documentation, and geospatially corrected based on comparison with species distribution maps from recent monographs on *Pteronia* (Kolberg and van Slageren, 2014; Bello, 2018). After these adjustments, 2 152 georeferenced occurrences were obtained for the species used in this study. To avoid discrepancies caused by uneven sampling, occurrence data were spatially stratified using the R package *spThin* (Aiello-Lammens et al., 2015) with a threshold distance of 10 km for each species. The occurrence data were then restricted to the area defined by the range of environmental data presented in Wüest et al. (2019). This approach led to a further reduction of available occurrence sites to 1 188 or 1 009 for calculation with soil database. These presence data were used to define a buffer area for collecting absence data according to recommendation, e.g. Warren et al. (2010). For this, an R package *geobuffer* (Valentin, 2022) and 10 km radius were used. Number of randomly chosen background points was set to hundred-fold of the presence occurrence points.

Our primary intention was to reveal possible correlation between TE and adaptation to the environment. Based on the results of the repetition analysis, we therefore classified the species of the genus *Pteronia* into two groups. One group contained species with significant TE abundance in their genomes compared to species with normal TE abundance in the other group. These groups were tested for differences in the environmental niche space occupied by the two groups of the genus *Pteronia*. We quantified overlap, equivalence, and niche similarity using an ordination technique that applies kernel smoothing to the presence of groups in environmental space using the *ecospat* R package (Broennimann et al., 2012; Di Cola et al., 2017). To interpret the niche characteristics, a PCA partition of the environmental space (first two axes) into a 500 × 500-cell grid was specified and the PCA output rasterized in this way was used to calculate niche overlap rates (derived from Schoener’s D statistic; Schoener, 1968), simulation tests of niche similarity and equivalence (Warren et al., 2008), and niche optimum and niche width estimates (Theodoridis et al., 2013; Kirchheimer et al., 2016; Duchoslav et al., 2021).

## Results

### Phylogenetic reconstructions based on the nuclear DNA low-copy genes and the chloroplast DNA dataset

**Figure 2:**
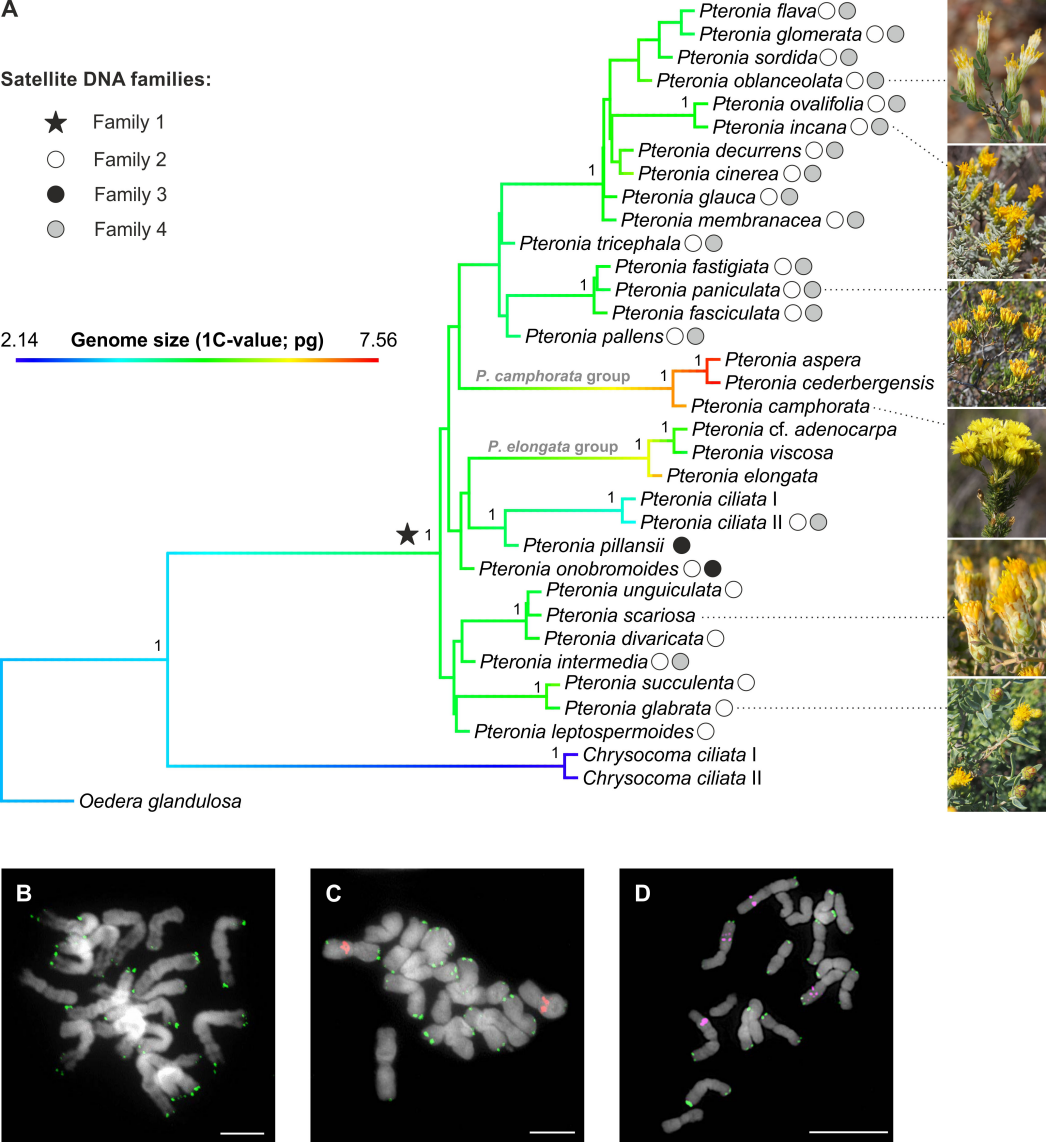
Phylogeny of the *Pteronia* species with detected families of satellite DNA. **(A)** ASTRAL species tree of the genus *Pteronia* based on 244 COS nuclear loci. The tree is rooted by two outgroups and the branches visualize the inferred evolution of genome size by reconstructing the ancestral state (using the contMap function in the *phytools* R package). Symbols at nodes and tips represent the main families of satellite DNA found in the studied species. Numbers at branches correspond to local posterior probabilities. **(B-D)** Chromosome localization of the satellite families 1 (green), 2 (red) and 4 (magenta) shown in *P. unguiculata* **(B)**, *P. ovalifolia* **(C)** and *P. incana* **(D)**. Chromosomes were counterstained by DAPI. Scale bars, 10 µm.

The presented species tree from the full nDNA dataset is based on 244 exonic loci (out of original 1 061 COS probes; **Figure 2**). Hyb-Seq statistics (e.g. total number of reads, mapped reads, missing data) are presented in **Supplementary Table 5** for each individual. The length of the concatenated plastome dataset after removing sites with more than 20% missing data was 146 998 bp and all statistics for individual species are presented in **Supplementary Table 5**.

The monophyly of *Pteronia* was strongly supported by the nuclear data in the ASTRAL species tree (1 LPP; **Figure 2**; **Supplementary Figure 2**) and by the plastome dataset in the ML tree (**Supplementary Figure 3**), but the internal structure of the ingroup is weak or virtually absent in both trees. In the nuclear species tree there are only two relatively well-supported clades that include species with high morphological and/or ecological similarity (group composed of *P. elongata* and relatives and group composed of *P. camphorata* and relatives; **Figure 2**, **Supplementary Figure 2**), whereas in the chloroplast tree even this weak structure is absent (**Supplementary Figure 3**). Discordance analysis of gene trees using PhyParts showed that almost all nodes are highly discordant, with only a small fraction of gene trees supporting a species tree, especially of the backbone nodes of the ingroup (**Supplementary Figure 2**).

Approximate date calibration of chloroplast tree estimated divergence of whole *Pteronia* clade to ca 3.99 Mya (CI = 3.68 – 4.33; **Supplementary Figure 3**).

### Flow cytometry

All 114 ingroup individuals (1–6 per population, **Supplementary Table 1**) and 4 outgroup individuals (one of *Oedera glandulosa* and three of one population of *Chrysocoma ciliata*) were subjected to flow cytometry. Clearly delimited peaks and low to moderate coefficients of variation (CVs; 1.11–6.74% for propidium iodide analyses; 1.00–3.31% for DAPI analyses), allowed for the precise estimation of their genome size and genomic GC content. Genome size in *Pteronia* samples varied 2.05-fold, ranging from 3.699 pg (3617.2 Mbp) in *Pteronia ciliata* to 7.578 pg (7411.7 Mbp) in *Pteronia cederbergensis* (**Figure 2**). The lowest genome size was estimated for outgroup *Chrysocoma ciliata* – 2.094 pg (2 047.9 Mbp). DNA base composition (GC content) did not differ much between taxa, with the lowest and highest values, *Pteronia glabrata* (59.0%) and *Pteronia membranacea* (61.7%), reaching only 2.7% difference. Summarized data are listed in **Supplementary Table 1**.

### Genomic traits evolution

**Figure 3:**
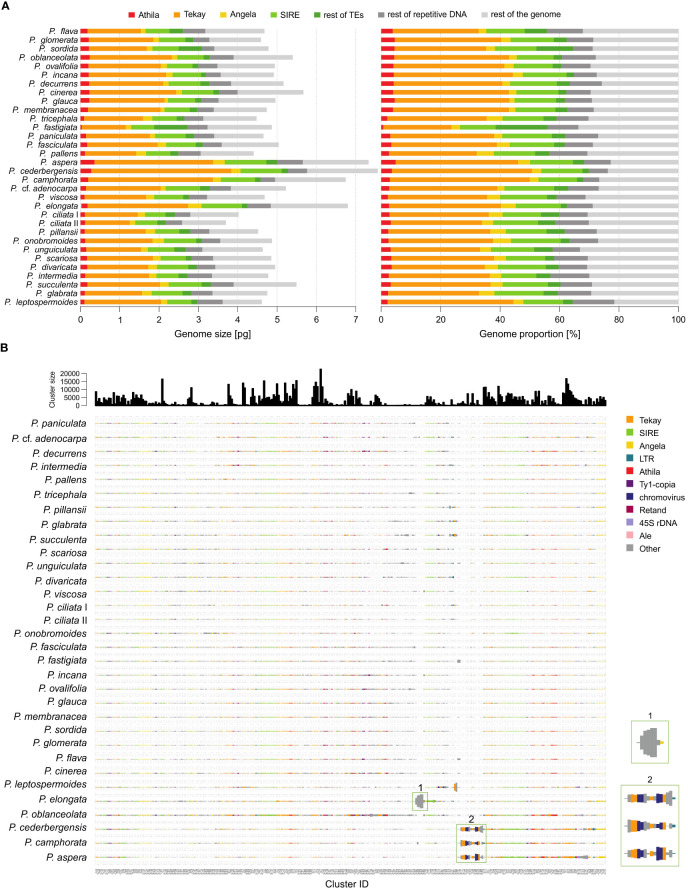
Repeat landscape of species of the genus *Pteronia*. **(A)** Bar graphs represent a graphical summary of the structure of the repeatome in phylogenetic order **–** the contribution of each category to genome size in absolute units in the left panel and in percentages in the right panel. Only the major categories involved in genome composition (mainly transposable elements of the Ty1-copia and Ty3-gypsy lineages) are shown. **(B)** Comparative analysis of genomes of 32 *Pteronia* species provided by RepeatExplorer. Bursts and putative bursts of TEs are highlighted and shown in the separate boxes on the right.

**Figure 4:**
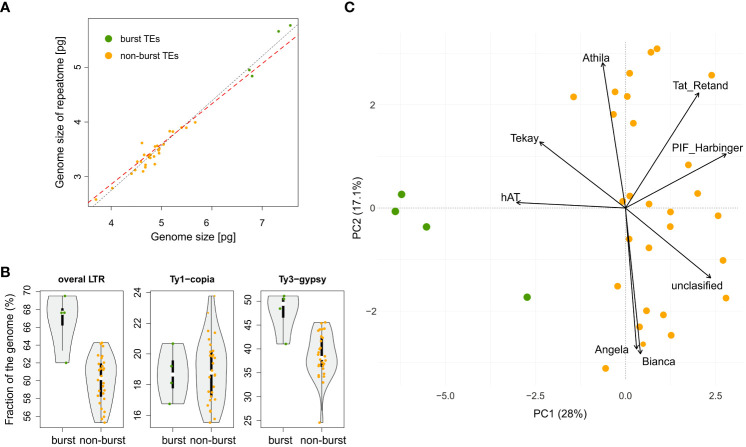
Contribution of repetitive genome components to genome size and delineation of two groups of species in the genus *Pteronia* **–** with and without TEs burst (burst – green dots vs non-burst – orange dots). **(A)** Relationship between whole genome size and genome size attributable to repetitive DNA. Black and red dashed lines represent linear models without and with correction for phylogeny, respectively. **(B)** Violin-plot of the major components of the repetitive genome (LTRs **–** long terminal repeats and their major lineages) and their contribution to the division of species in the genus *Pteronia*. **(C)** PCA ordination of the repeatome components and the components that contribute most to the delimitation of the two groups of species in the genus *Pteronia*.

The BM model was inferred as the best approximation of genome size evolution (**Table 1**). The only improvement against the BM evolution model was ascribed to the δ model that estimates change of trait evolution in time (δ = 2.361, 95% CI = 1.603, 2.985). The level of δ > 1 indicates greater changes in the rate of trait evolution closer to the present. Despite an overall tendency to retain medium-sized genomes, evidence of genome downsizing is apparent in some species, especially in *Pteronia ciliata*. The opposite trend of genome expansion occurs in other lineages, such as the *Pteronia aspera*, *P. cederbergensis* and *P. camphorata* group, and the same tendency is apparent also in *Pteronia elongata* (**Figure 3**). Such changes are apparently linked to gain or loss of transposable elements (**Figure 4**). The correlation analysis revealed a tight association between overall genome size and genome size associated with repeatome (simple correlation – R^2^ = 0.986, *p* < 0.001, correlation with phylogenetically independent contrast – $R_{\text{pic}}^{2}=0.936$ , *p*_pic_ < 0.001). Obviously, the tight correlation is mainly driven by proliferation of LTR (simple correlation – R^2^ = 0.982, *p <* 0.001; $R_{\text{pic}}^{2}=0.923$ , *p*_pic_ < 0.001). Contrary, there were no clear trends in GC content in the genus *Pteronia*, which could be attributed to the lack of considerable variability (data not shown).

**Table 1:** Models of trait evolution for genome size and their superior fit compared to the basic BM (Brownian motion) model.

model	AICc	LogLM	Estimated parameter		Comparison to BM model	
			value	95% CI	ΔAICc	p-value
BM	56.805	-26.196	–	–	–	–
lambda	58.335	-25.739	λ = 0.89	0.54 – 1.00	-1.53	0.499
delta	48.457	-20.800	δ = 2.36	1.60 – 2.98	**8.35**	**0.020**
kappa	59.249	-26.196	κ = 1.00	0.56 – 1.00	-2.44	1.000
OU	59.196	-26.169	α = 0.09	0.00 – 1.14	-2.39	0.871

Significantly better model than BM model is in bold.

### Chromosome counts

Chromosome counts were determined in 16 of 32 ingroup *Pteronia* samples (**Supplementary Table 1**) for which germinated seedlings were available. Mitotic chromosome spreads from root-tips revealed diploid chromosome number 2*n* = 18 in 14 accessions. An uncertain chromosome count around 18 was observed in the two remaining accessions, and this was probably caused by fragile satellites (**Supplementary Figure 4**).

### Repeatome composition

The RE pipeline used an average of 1.35 million paired reads with no overlap per sample, corresponding to a genomic coverage of 0.05–0.10×. Based on the RE analysis, it was found that the investigated species differ in the structure of the repeats. **Supplementary Figure 1** shows a graphical summary of the clustering results for selected genomes of the genus *Pteronia* and the average data from the RE comparative analysis, which belong to different lineages according to the phylogenetic analysis. The *Pteronia* genomes are enriched with LTR retrotransposons constituting from 39.5% (*P. fastigiata*) to 68.9% (*P. cederbergensis*) of the genome, with predominance of Ty3-*gypsy* chromovirus lineage, *Tekay* clade (up to 47.1%), and Ty1-*copia* element of *Maximus/SIRE* clade (up to 16.7%) (**Figure 4**, **Supplementary Table 2**). The amount of LINE and DNA elements are less than 0.5%.

Comparative analysis of 32 *Pteronia* species revealed several TE bursts events. In the genome of *P. elongata*, clusters 248, 257, 294, 306 and 330 show a significant excess of copy-number compared to other species (**Figure 3**). In these clusters a small percentage of *Tekay*-related reads were determined, and they are definitely a disintegrated residue of *Tekay* elements. More definite bursts of *Tekay* elements were observed in genomes of *P. aspera*, *P. cederbergensis* and *P. camphorata* where clusters 38, 39, 122, 144, 168, 171, 195, 224, 245, 296 and 320 show a significant excess of copy-number compared to other species (**Figure 3**). Among these clusters, four were defined as *Tekay* element (in these clusters all *Tekay* retrotransposon conserved domains namely GAG, INT, RT, RH, PROT and CHD were present), four as unidentified chromovirus and the rest are of unknown origin. In addition, clusters 190 and 274, which also showed a significant (approximately 10-fold) copy number excess compared to the other species, were defined as belonging to the Ty1-*copia* retrotransposon of the TAR clade. The contribution of main TEs superfamilies to genome size variation is given in **Supplementary Table 2** and in **Figure 4** that simultaneously show the differences between species with bursted and non-bursted TEs.

PCA of the entire TEs genomic pool confirmed bursts of TEs previously identified by comparative TE analysis in genomes of *P. aspera*, *P. camphorata*, *P. cederbergensis* and *P. elongata* by separating these species in the PCA space (**Figure 4**).

### RE analysis determined four major satDNA families in genomes of investigated *Pteronia* species

Application of the RE pipeline clustering tool for Illumina reads of genomes of thirty-two diploid *Pteronia* species resulted in the identification of four major satDNA families that were determined in two or more species (i.e. potentially group-specific; **Figure 2**, **Supplementary Table 2**). SatDNA families (i.e. shared monomers with conserved motifs), with consensus monomers less than 300 bp and for which the BLAST search produced zero results, were taken into account. By this, fragments and decaying LTR TEs with which genomes of *Pteronia* are enriched were excluded. Basic characteristics of detected satDNA families are given in **Supplementary Table 3**. *In silico* scanning of the assembled genomes at the contig level revealed that family 1 is present in the genomes of all species studied, and FISH analysis placed it in subtelomeric regions of the chromosomes (**Figure 2**). Family 2 was present in the genomes of twenty-three species (**Figure 2**, **Supplementary Table 2**) in the pericentromeric regions of one or two chromosome pairs (**Figure 2**). Family 3 was present in the genomes of only two species, *P. onobromoides* and *P. pillansii* and was not reliably detected by FISH probes. Family 4 was present in the genomes of seventeen species (**Figure 2**, **Supplementary Table 2**) on two pairs of long chromosomes in the middle of long arms as interstitial loci (**Figure 2**). Consensus monomers of the satDNA families were less similar to each other than 35%.

### Niche modelling and biome preferences

**Figure 5:**
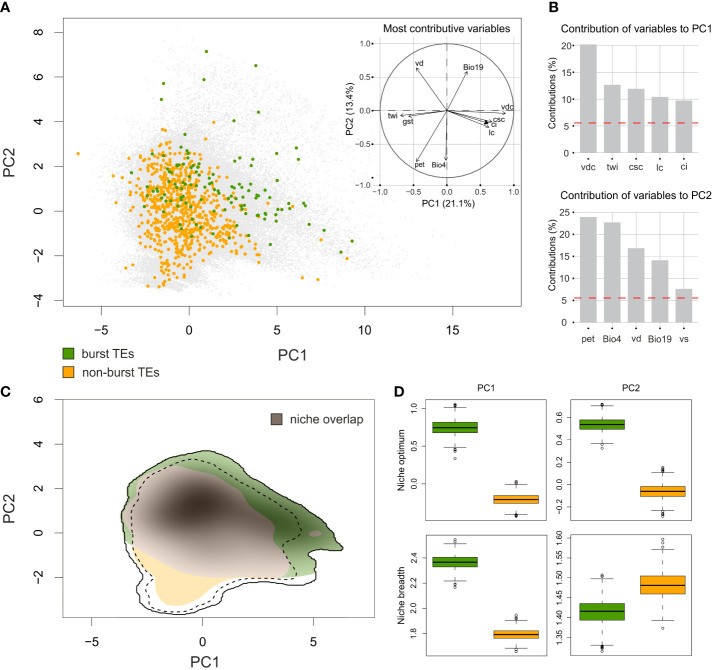
Environmental niche modelling of *Pteronia* species based on CHELSA Bioclim data and additional extra-CHELSA and topographic data taken from Wüest et al. (2019). **(A)** PCA ordination performed by *ecospat* R package based on background points (gray) and the position of sampling points of species with burst TEs (green) and non-burst TEs (orange). The inset shows the contribution of main environmental characters to the first two PCA axes. **(B)** Percentage contribution of the five most important environmental characters for the first two PCA axes. **(C)** Comparison of niches occupied by two groups of *Pteronia* species with different TEs composition of their genomes. Shaded colors follow the common pattern and putative niche overlap is in gray. Full and dashed contour lines illustrate 100 and 75%, respectively, of available environments delimited by a 10-km buffer zone around the occurrence points of each *Pteronia* group. **(D)** Niche optima (top two panels) and niche breadths (bottom two panels) for both *Pteronia* groups along the first (left) and second (right) PCA axes. Explanation of abbreviations: vdc – vertical distance to channel network, twi – topographic wetness index, csc – cross-sectional curvature, lc – longitudinal curvature of the terrain, ci – convergence index, pet – potential evapotranspiration, Bio4 – temperature seasonality, vd – valley depth, Bio19 – precipitation of coldest quarter, vs – visible sky.

The variation in the environmental conditions in the target area was summarized by two PCA axes, explaining 21.1% and 13.4%, respectively, of the total variation in the environmental space (**Figures 5A**). The PC1 axis was strongly associated with topographic variables derived from the digital elevation model and extra CHELSA characteristics. The most contributive variables mirrored the terrain heterogeneity and complexity and the water availability (vertical distance to channel network (vdc), topographic wetness index (twi), cross-sectional curvature (csc), longitudinal curvature of the terrain (lc) and convergence index (ci)). The PC2 axis was associated with variables that reflect water regime, terrain characteristics and temperature amplitudes (potential evapotranspiration (pet), temperature seasonality (Bio4), valley depth (vd), precipitation of the coldest quarter (Bio19) and visible sky (vs)). See **Supplementary Table 4** for detailed description of the variables and their contributions to PCA axes. Both groups of *Pteronia* species, as defined by TEs amplification, apparently overlapped in their environmental demands (**Figure 5**), represented by the moderate value of the Schroner’s D metric (0.347). However, the simulation test for niche similarity revealed no evidence of niche conservatism or divergence (*p* = 0.38 and *p* = 0.65, respectively). Contrary, analysis of niche optimum pointed to high level of divergence between the two groups (**Figure 5**). *Pteronia* species with bursted TEs tend to occupy places with lower potential evapotranspiration, higher precipitation in the coldest as well as the warmest quarter of the year, higher vertical distance to the level of groundwater, lower mean temperature of all growing season days, smaller index of isothermality, etc. (**Supplementary Figure 5**). Niche breadths of both groups are almost equal, but are apparently shifted in environmental space along both PCA axes (**Figure 5**). Very similar results, despite the limited occurrence data, were obtained using environmental data with soil. Although the overall overlap was higher and the difference between the two groups less pronounced, their separation from each other in both niche optima and niche breadth remained (**Supplementary Figure 6**).

Species occurrence data were also used to estimate preferences in occupancy of major biomes within the GCFR. Although in some cases the availability of these data was limited (rare species with fewer than five known occurrences), we estimated at least rough biome preferences for 24 of the 31 taxa in the genus *Pteronia* (**Supplementary Figure 7**). Some species exhibit a wide range of biomes where they can grow, while other species dominate or are restricted to a single biome. All four species with burst TEs are closely tied to Fynbos and their occurrence in other biomes is sporadic (or doubtful due to the inability to reassess recorded occurrence data).

## Discussion

Genus *Pteronia* represents an interesting distinct evolutionary lineage within the tribe Astereae (e.g. Brouillet et al., 2009; Mandel et al., 2019) with a center of diversity and high level of endemism in the GCFR (Manning and Goldblatt, 2012; Snijman, 2013). In the course of thorough investigation of this genus, we have found a high level of genome size variability associated both with heteroploid and homoploid variation. Here we investigate the causes and consequences of variation in homoploid genome size at the diploid level within a phylogenetic framework.

### Hyb-Seq phylogeny in rapidly radiated plant groups

Relatively stringent filtration resulted in a relatively low number of exonic loci, on the other hand we obtained a dataset with high completeness and quality. In *Pteronia* we observed a similar pattern as in other recent works using COS probes of Mandel et al. (2014) on a recently radiating group (e.g. Gizaw et al., 2022; Kandziora et al., 2022), i.e. relatively short branch lengths in some parts of in-group phylogenetic tree topology, which is probably related to incomplete lineage sorting (ILS) and possible hybridization. Also, due to ILS we observe medium support and short branch lengths on some internal nodes of the ASTRAL species tree. The probes were designed for the whole Asteraceae family (Mandel et al., 2014), so lower resolution within a single genus is to be expected. A possible solution would be to design custom probes, but it would require at least a draft assembly of the *Pteronia* genome, and the relative benefit over generic probes seems to lie in data completeness rather than increased phylogenetic informativeness (Ufimov et al., 2021). Besides, even comparison of nearly complete chloroplast sequences shows no differentiation in species of the genus *Pteronia* (**Supplementary Figure 3**). Time calibration applied to chloroplast phylogeny further shows relative recent divergence within the *Pteronia* clade (~ 4 Mya), even though its divergence from the rest of Asteraceae probably happend much earlier (~ 12 Mya, Mandel et al., 2019). It likely points to the existence of some time-limited event that triggered a radiation within *Pteronia*. The exact timing of the detected fast radiation is a matter for further research and would need to be compared with significant environmental changes in the area (inspired by e.g. Kong et al., 2021). However, evidence from species-rich plant lineages from the GCFR, and particularly from Succulent Karoo flora, suggests rapid speciation events triggered by Miocene–Pliocene climatic change (e.g. Dupont et al., 2011). This climatic change has been associated with the development of a summer-arid climate, which noticeably affected the flora of the northern and western GCFR and caused the local lowland flora to transition to a semi-arid succulent vegetation (Verboom et al., 2014). These authors further suggest that, assuming that the onset of seasonal drought accelerated the replacement of mesic flora (i.e., fynbos or forest) by drought-adapted flora (as hypothesized by Levyns (1964)), the succulent karro flora can be expected to be dominated by young, drought-adapted lineages. This evolutionary pattern has been inferred for some of the major clades of Cape plants (e.g., Klak et al., 2004; Verboom et al., 2009). We can speculate that a similar pattern of speciation took place in *Pteronia* and was triggered by the onset of a summer and dry climate at the end of the Miocene (i.e., ~5 Mya).

### Repeatome-related evolutionary events

Application of the RE pipeline for analysis of whole genome shotgun Illumina reads from the genomes of thirty-two diploid *Pteronia* South-African endemic species from divergent lineages revealed several evolutionary events in the repeatome components (**Figures 2**, **3**).

(1) *Bursts of TE.* A burst of TEs is a massive outbreak that may cause radical genomic rebuilding. This phenomenon has been reported in connection with the formation of taxonomic groups and species and has therefore been associated with major evolutionary events in the past (Belyayev, 2014). We found a very clear increase in copy-number of *Tekay*, unidentified chromovirus and *TAR* LTR retrotransposons in genomes of *P. aspera*, *P. camphorata* and *P. cederbergensis* that belong to the separate lineage according to phylogenetic analysis. An earlier TE burst occurred also in the *P. elongata* genome but we could not identify the TEs precisely, most probably due to the rapid post-burst disintegration of involved TEs (Liu & Wendel, 2000). Nevertheless, some of the reads in the increased clusters were defined as *Tekay*-related.

(2) *satDNA depletion*. In genomes of the species with putative bursts of TEs, the tandem repeats of Families 2, 3 and 4 (see below) were completely absent. This can be explained by the burst-purification cycles mechanism when a certain number of TE copies is “cleaned” from the genome thus equilibrating TE copy-numbers (for review see Le Rouzic and Deceliere, 2005). Ectopic recombination that is the main background force for TEs reduction (Devos et al., 2002) may encompass not only a TE fraction but also the entire amount of highly repetitive DNA (Belyayev, 2014). The majority of the TEs are located in heterochromatin (Heslop-Harrison et al., 1997; Belyayev et al., 2001; Saunders and Houben, 2001) and closely located sequences of other types could also be eliminated. Such a model for depletion of tandem repeats as a result of TEs burst-purification cycles was suggested in marginal populations of *Aegilops speltoides* Tausch (Poaceae, Monocotyledons; Raskina et al., 2011). We can speculate whether our data, from evolutionary distant *Pteronia* (Asteraceae, Dicotyledons) genomes, resemble a similar phenomenon, albeit at the species level.

(3) *Origin of group-specific tandem repeats.* The presence-absence of a certain family of tandem repeats in the genome is a strong indicator of the species relatedness. Among the four major satDNA families determined in genomes of *Pteronia*, Family 1 is present in all investigated species which confirms their common origin. This satDNA family most probably formed at the initial stage of the evolution of the group (**Figure 2**). Family 2 was also apparently indigenous for all species, but its elimination may have occurred due to TE bursts in the genomes of *P. aspera*, *P. camphorata*, *P. cederbergensis* and *P. elongata*, and in genomes of *P.* cf. *adenocarpa, P. ciliata, P. pillansii, P. scariosa*, and *P. viscosa* for unknown reasons. Family 3 was formed only in two species, *P. onobromoides* and *P. pillansii*, that early diverged from the main group, although *P. onobromoides* possesses Family 2 repeats and *P. pillansii* not. Family 4 aggregates a large group of closely related species with the exception of *P. ciliata* II and *P. intermedia* that are distantly placed (**Figure 2**). Interestingly, the presence of Family 2 and 4 of satDNA also distinguishes two specimens attributed to *P. ciliata* that are closely related but also differ in genome size (**Figure 2**, **Supplementary Table 1**).

We can summarize that the average repeatome of *Pteronia* genomes is fairly typical for Asteraceae (Staton and Burke, 2015). The *Tekay* chromovirus family as the main component of the dominating Ty3-*gypsy* LTR retrotransposons is consistent with some other members of the family Asteraceae, such as the genus *Marshallia* Schreb. (Hall and Goertzen, 2016) and *Hieracium* L. (Zagorski et al., 2020). On the other hand, variations were found in other representatives, such as the genus *Anacyclus* L. (subfamily Asteroideae), where SIRE lines from the Ty1-*copia* retrotransposon LTR superfamily emerged as the most dominant TE group (Vitales et al., 2020). In general, the proportion of the genome that is composed by various TE superfamilies is very much in line with the phylogenetic position of the genus *Pteronia* within the family Asteraceae according to Staton and Burke (2015). They revealed an increasing proportion of Ty3-*gypsy* and a decreasing proportion of Ty1-*copia* TEs along the Asteraceae phylogeny (in the direction from basal to advanced lineages). Intrageneric bursts of TEs are in *Pteronia* manifested mainly by multiplication of LTR retrotransposons, especially by dominant chromoviral clade *Tekay* (**Supplementary Table 2**). This contrasts with the finding in *Hieracium*, where co-growing *Tekay* and SIRE modulate genome size variation (Zagorski et al., 2020). The magnitude of transpositional bursts in the taxa under investigation corresponds directly with genome size variation on diploid level (**Figure 2**). The same pattern and a similarly tight correlation has been elucidated, for example, in diploid lineages of the genus *Helianthus* L. (Asteraceae, Tetreault and Ungerer, 2016). We further found that bursts of TEs may have been responsible for the elimination of some major satDNA families, thus resulting in a new repeatome landscape. Since TEs bursts can cause reproductive isolation between populations (Belyayev, 2014), e.g. by altering chromosome segregation (Ferree and Barbash, 2009), it is plausible that they can cause the emergence of separate lineages at the species level, as is partly indicated by the *Pteronia* phylogenetic analysis. Given the weak phylogenetic differentiation of the genus due to the rapid radiation, repeatome analysis appears to be a useful tool to distinguish some lineages on a molecular basis. The rate of TE accumulation and the associated genome rearrangement seem to contribute significantly to a good separation of only those lineages that also underwent TE bursts. One such example is the isolated lineage of three species of the genus *Pteronia* (*P. aspera, P. camphorata and P. cederbergensis*), which also represents a morphologically and ecologically distinct group within the genus (Bello et al., 2017).

### Ecological implications of TEs burst

Based on repetitive DNA analysis, we were able to distinguish two main groups of species within the studied genus *Pteronia* – species with average and expected TEs multiplication rates relative to other members of the family Asteraceae (non-burst) and species with significantly increased TEs multiplication rates (burst). Interestingly, the occurrence of all four identified species with burst TEs is restricted to the Fynbos biome in the Cape Floristic Kingdom (**Figure 1**), which covers a shrubland vegetation with a huge species diversity and is characterized by e.g. very low soil nutrient content, periodic fires and dominance of winter rains (reviewed by Allsopp et al., 2014). Therefore, the ecological status of these species in relation to the other (non-burst) species was tested to evaluate the plausible contribution of TEs proliferation to ecological differentiation or vice versa.

Our data suggest that the environmental niches of the two groups of species are somewhat similar and overlap, yet their optima and breadths are considerably shifted (**Figure 5**). The phenomena that may play a role in these changes are numerous, as TEs are thought to significantly modulate gene expression by repressing or enhancing regulatory functions or inducing stress responses (reviewed e.g. by Lisch, 2013; Bennetzen and Wang, 2014; Dubin et al., 2018). A generally admitted scenario involves stress conditions or environmental stimuli early on that trigger bursts of TEs that sometimes cause genomic changes leading to rapid adaptation to the new environmental conditions (Dubin et al., 2018). Although we can only speculate whether this also happened in *Pteronia*, the solitary and relatively distant lineage comprising *P. aspera*, *P. camphorata* and *P. cederbergensis* (Bello et al., 2017) indicate strong environmental differentiation, which is also associated with a pronounced TE burst with a specific pattern (**Figure 3**). A similar, yet different in output, scenario is likely to have taken place in *P. elongata*, which represents a somewhat isolated diploid *Pteronia* in terms of TEs burst pattern (**Figure 3**) and phylogenetic position. It appears to be more closely related to derived polyploid lineages such as *P. hutchinsoniana* Compton and *P. stoehelinoides* DC. (not included in this study) than other diploid species, however, both polyploids are known to occur outside Fynbos, but this needs further investigation. Thus, ecological transitions to an exclusively Fynbos biome seem to induce a small radiation in species numbers at least in the first case. This is consistent with the findings of Bouchenak‐Khelladi and Linder (2017), who speculate on the very important role of local environmental transitions in shaping the vast plant diversity of the GCFR. Although our data suggest that both transitions in *Pteronia* are associated with burst TEs, the question remains as to what was the primary cause and what was the consequence.

## Data availability statement

The data (raw sequence reads) presented in the study are deposited in the NCBI repository as Sequence Read Archive (SRA) BioProjects under accession number PRJNA821402 and PRJNA821405 for target enrichment (Hyb-Seq) data, and PRJNA821007 for shallow whole-genome sequencing (repeatome and cpDNA phylogeny) data.

## Author contributions

ZC and PT conceived and designed the study. ZC, KS, DE-B and PT sampled plant material. ZC, AB, TM, KS, VZ, EH and PT collected and analyzed the data. ZC, AB and PT drafted the manuscript with the contribution of TM and VZ. All authors contributed to the article and approved the submitted version.

## Funding

The study was financed by the Czech Science Foundation (project no. 19-20049S) and the Ministry of Education, Youth and Sports of the Czech Republic, Operational Programme Research, Development and Education, The European Structural and Investment Funds, EU (project no. CZ.02.2.26/0.0/0.0/16_027/0007852). Additional support was provided as part of a long-term research project of the Czech Academy of Sciences, Institute of Botany (RVO 67985939). Computational resources were supplied by the project “e-Infrastruktura CZ” (e-INFRA CZ LM2018140) supported by the Ministry of Education, Youth and Sports of the Czech Republic.

## Acknowledgments

We thank Dora Čertnerová, Martin Čertner and Jan Ptáček for help with fieldwork, Lenka Flašková for her help in the laboratory, Roswitha E. Schmickl for her valuable advice in phylogenetic analyses and Karol Krak for valuable comments on the first version of the manuscript. We are indebted to Blanka Hamplová and Štěpánka Hrdá from the BIOCEV OMICS Genomika center (Vestec, Czech Republic), to the employees of the Institute of Applied Biotechnologies (Olomouc, Czech Republic) and the Admera Health Biopharma Services (New Jersey, USA) for their help with sequencing. We thank Nicola Bergh (NBG) and Cornelia Klak (BOL) for their friendly approach during our visits to the herbaria and to Léanne Dreyer for all her help and especially for providing space for a flow cytometer in her laboratory at the Stellenbosch University during a six-month internship of ZC. Cape Nature is acknowledged for providing collecting permits.

## Conflict of interest

The authors declare that the research was conducted in the absence of any commercial or financial relationships that could be construed as a potential conflict of interest.

## Publisher’s note

All claims expressed in this article are solely those of the authors and do not necessarily represent those of their affiliated organizations, or those of the publisher, the editors and the reviewers. Any product that may be evaluated in this article, or claim that may be made by its manufacturer, is not guaranteed or endorsed by the publisher.
