# Supplementary material for: The relationship between transposable elements and ecological niches in the Greater Cape Floristic Region: A study on the genus Pteronia (Asteraceae)
Source: Front Plant Sci. 2022 Sep 29;13:982852. doi: 10.3389/fpls.2022.982852 (PMC9559566; doi:10.3389/fpls.2022.982852)
Supplement: Supplementary file 1 [file DataSheet_1.pdf]

Supplementary Material

The relationship between transposable elements and ecological niches in the Greater Cape Floristic Region: a study on the genus *Pteronia* (Asteraceae)

Zuzana Chumová, Alexander Belyayev, Terezie Mandáková, Vojtěch Zeisek, Eva Hodková, Kristýna Šemberová, Douglas Euston-Brown, Pavel Trávníček

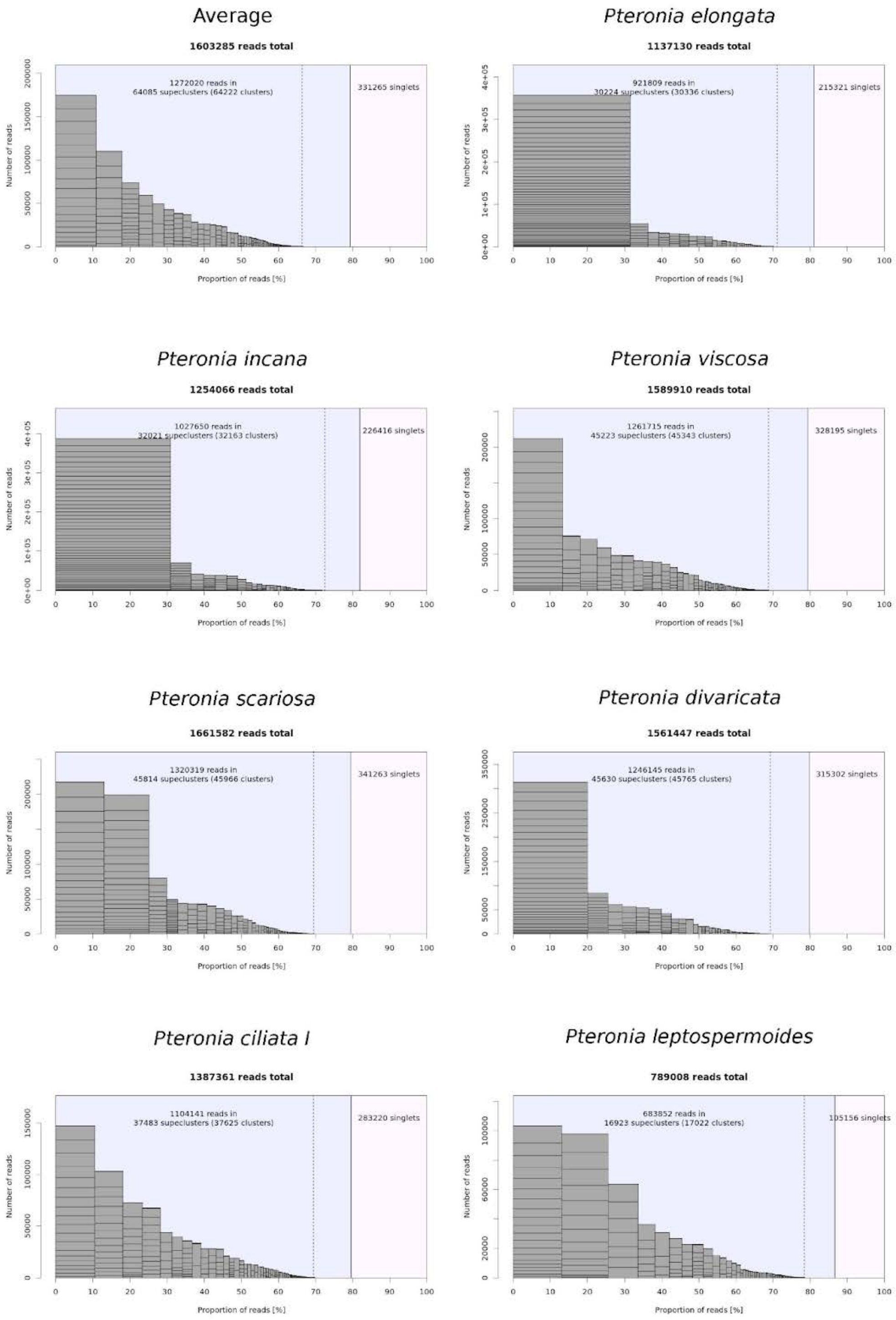

Supplementary Figure 1 | Graphical summary of the repeatome structure. Bars represent superclusters, with their heights and widths corresponding to the numbers of reads in the superclusters (y-axis) and to their proportions in all analyzed reads (x-axis), respectively. Rectangles inside the supercluster bars represent individual clusters.

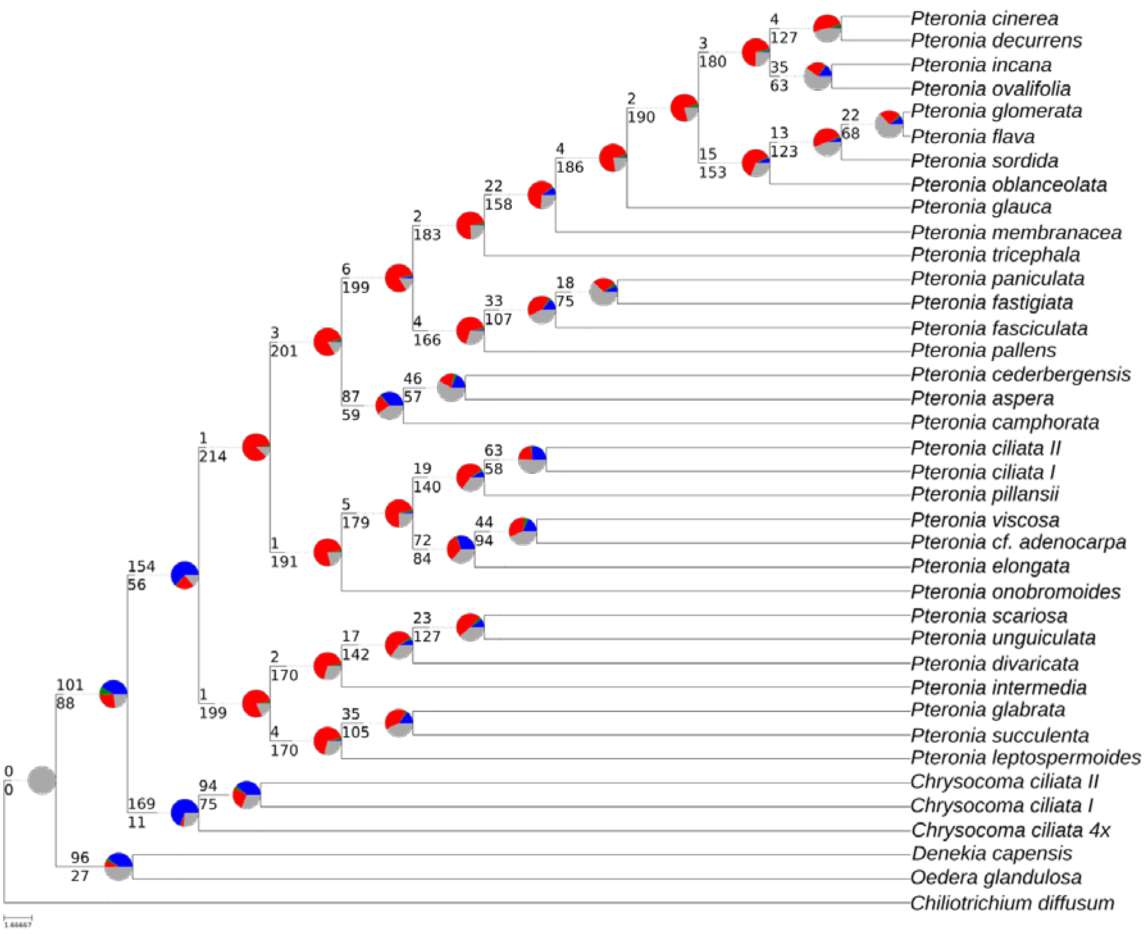

Supplementary Figure 2 | Species tree topology obtained via the ASTRAL approach of the genus *Pteronia*, based on 244 nuclear low-copy genes. For each branch, the top number indicates the number of gene trees concordant with the species tree at that node, and the bottom number indicates the number of gene trees in conflict with that clade in the species tree. The pie charts at each node present the proportion of gene trees that support that clade (blue), the proportion that support the main alternative for that clade (green), the proportion that support the remaining alternatives (red), and the proportion that inform (conflict or support) this clade that have less than 50% bootstrap support (grey).

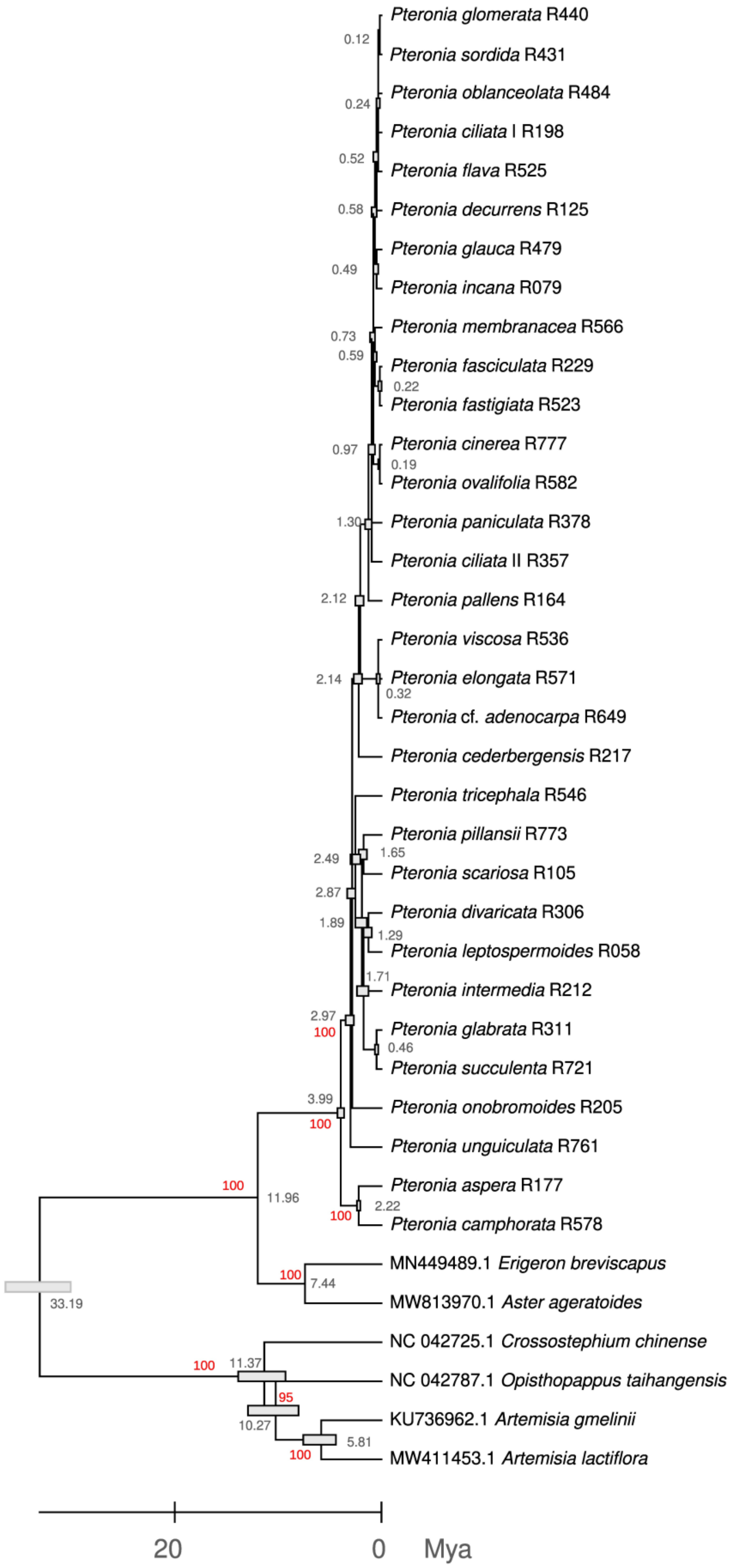

Supplementary Figure 3 | Time calibrated ML tree topology based on cpDNA data (146 998-- bp) with bootstrap support for main branches. Due to low differentiation and generally short branches, support is omitted even for several branches reaching 100%.

Chromosome counts in *Pteronia*

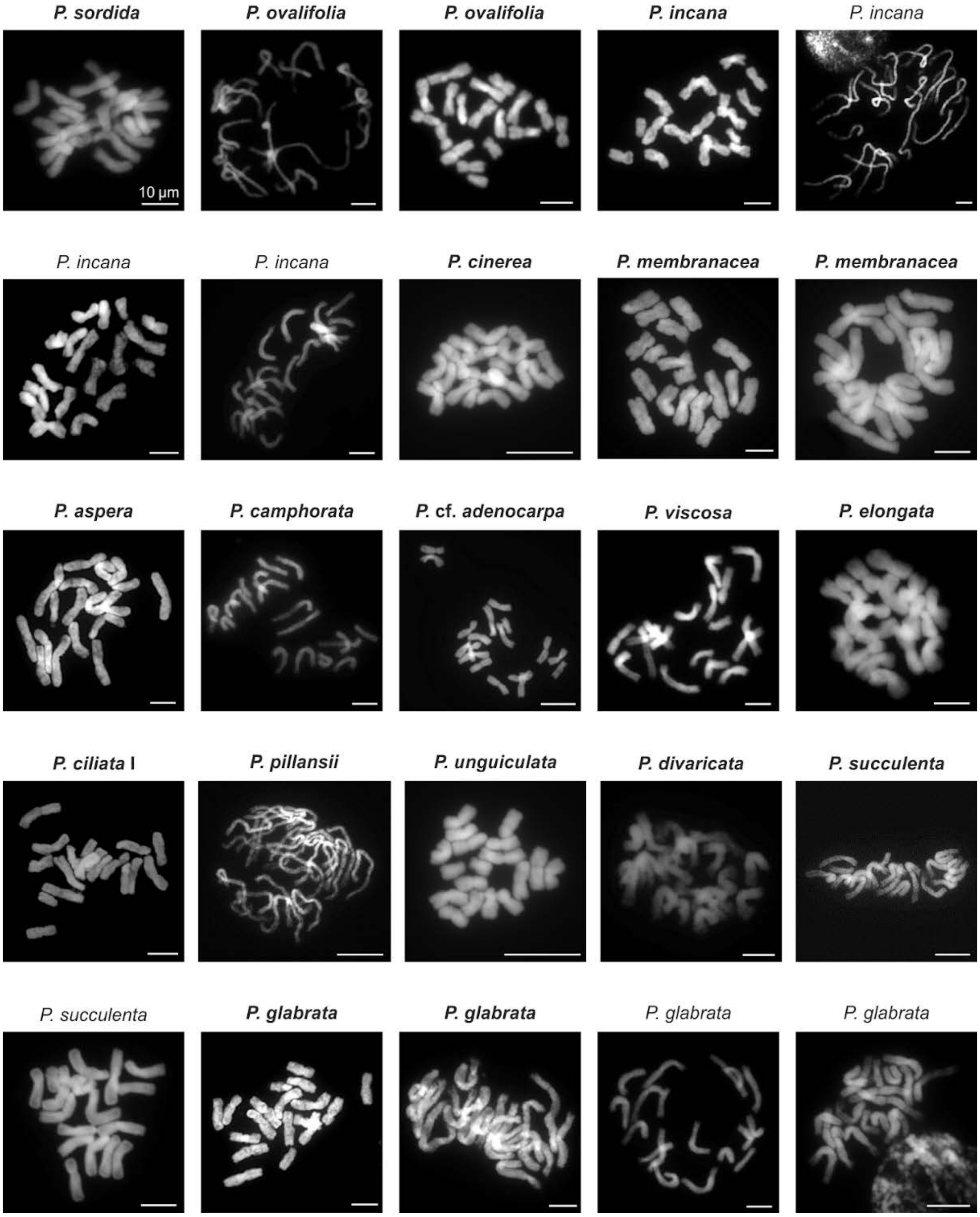

Supplementary Figure 4 | Mitotic chromosome preparations of 25 diploid *Pteronia* accessions (all 2n = 18). Chromosomes were counterstained by DAPI. Scale bars, 10 um.

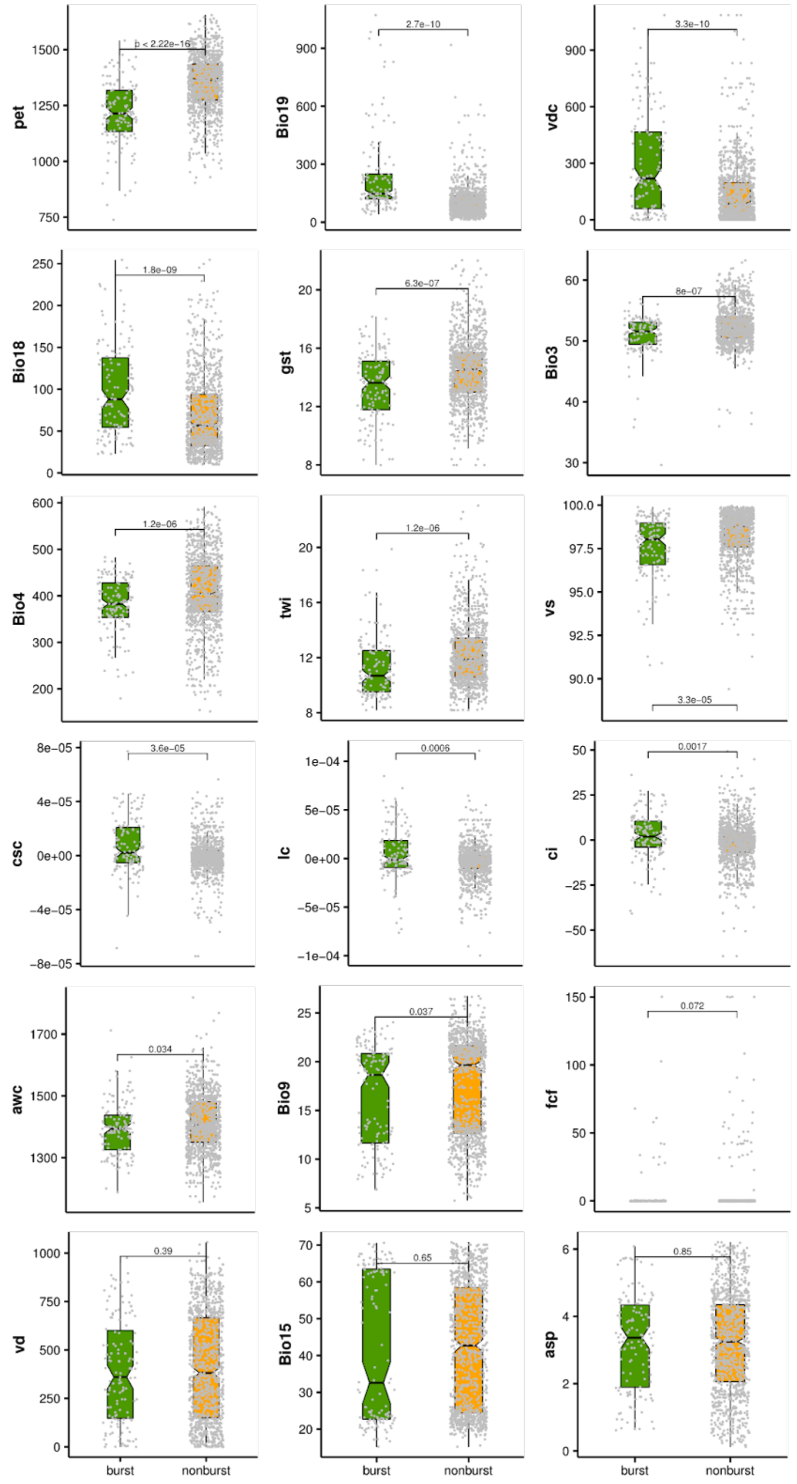

Supplementary Figure 5 | Overview of all environmental variables used for niche analysis of *Pteronia* species divided into two groups based on TEs bursts (green = burst, orange = non-burst). Boxplots are supplemented with *p*-value of t-test and the variables are ordered by its increasing value. See Table S4 for a brief description of the variables.

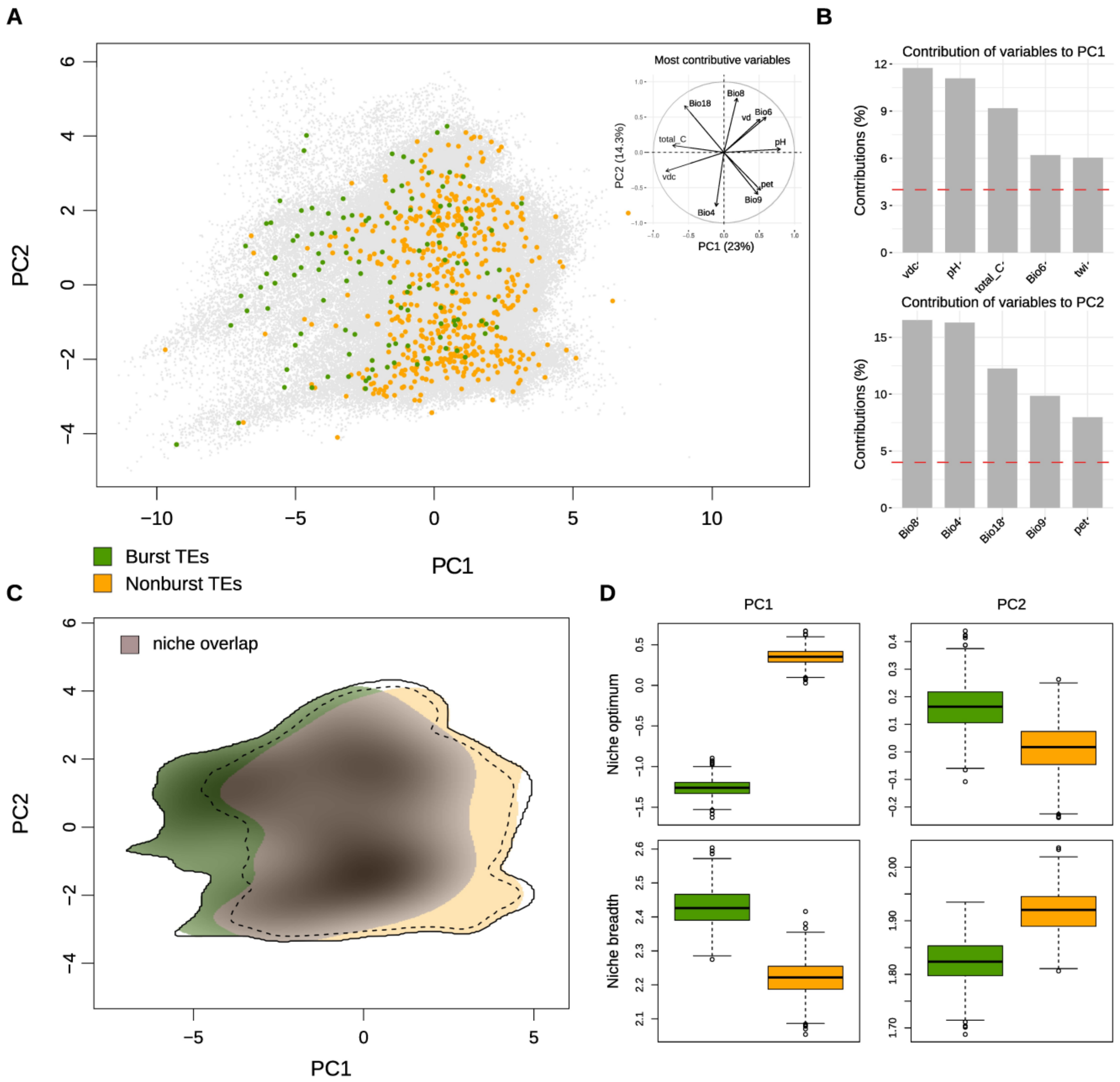

Supplementary Figure 6 | Environmental niche modeling of *Pteronia* species based on CHELSA Bioclim data, additional extra-CHELSA and topographic data taken from Wüest et al. (2019), and soil data taken from Cramer et al. (2019). (A) PCA ordination performed by ecospat R package based on background points (gray) and the position of sampling points of species with burst TEs (green) and non-burst TEs (orange). The inset shows the contribution of main environmental characters to the first two PCA axes. (B) Percentage contribution of the five most important environmental characters for the first two PCA axes. (C) Comparison of niches occupied by two groups of *Pteronia* species with different TEs composition of their genomes. Shaded colors follow the common pattern and putative niche overlap is in gray. Full and dashed contour lines illustrate 100 and 75%, respectively, of available environments delimited by a 10-km buffer zone around the occurrence points of each *Pteronia* group. (D) Niche optima (top two panels) and niche breadths (bottom two panels) for both *Pteronia* groups along the first (left) and second (right) PCA axes.
Explanation of abbreviations: vdc – vertical distance to channel network, twi – topographic wetness index, csc – cross-sectional curvature, lc – longitudinal curvature of the terrain, ci – convergence index, pet – potential evapotranspiration, Bio4 – temperature seasonality, vd – valley depth, Bio19 – precipitation of coldest quarter, vs – visible sky.

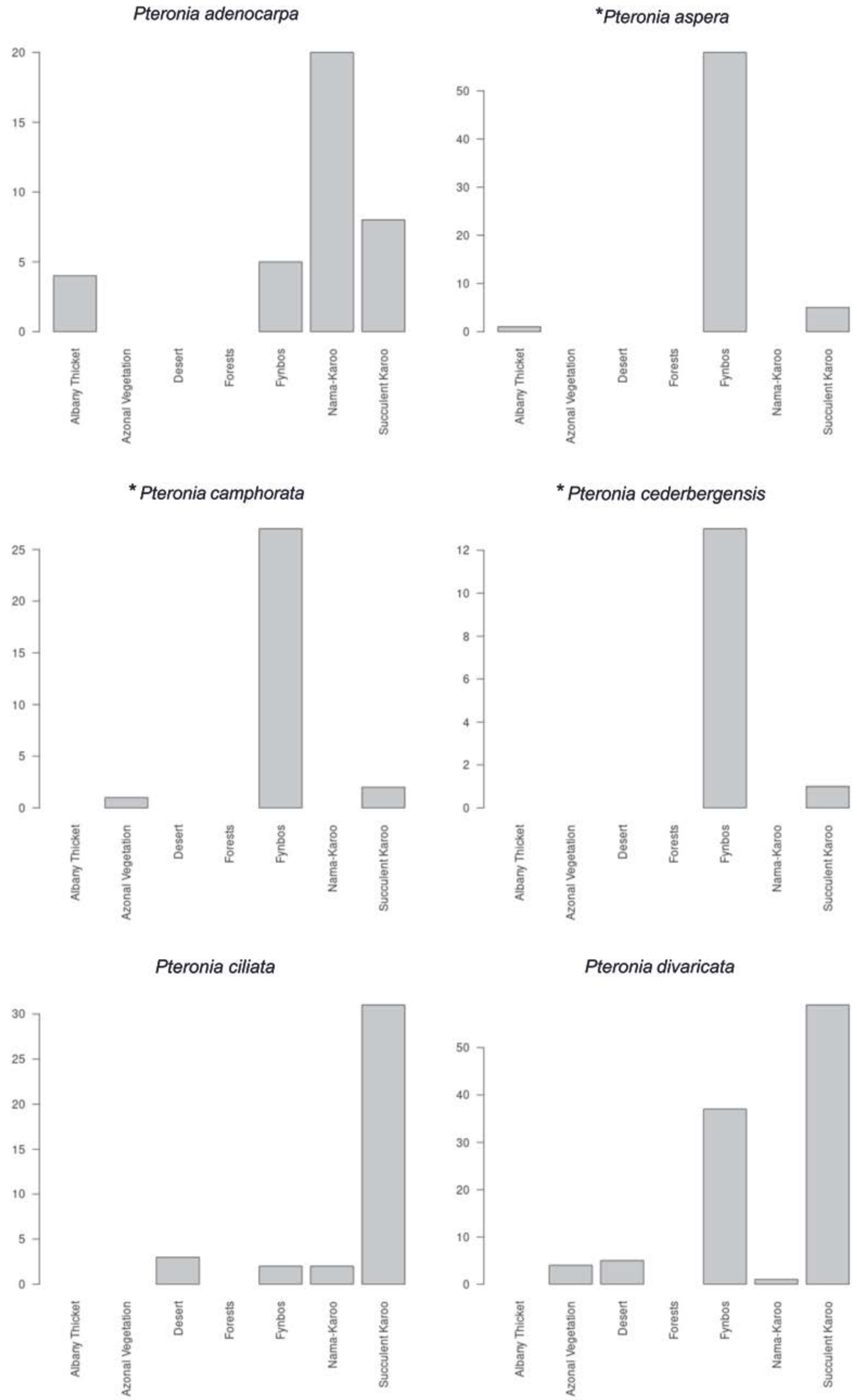

Supplementary Figure 7 | Bar graphs showing the association of 24 species of the genus *Pteronia* with major biomes in South Africa. The species presented were accompanied by sufficient occurrence data to allow at least a rough assessment of association with biomes. Species names marked with asterisks indicate species with TEs burst.

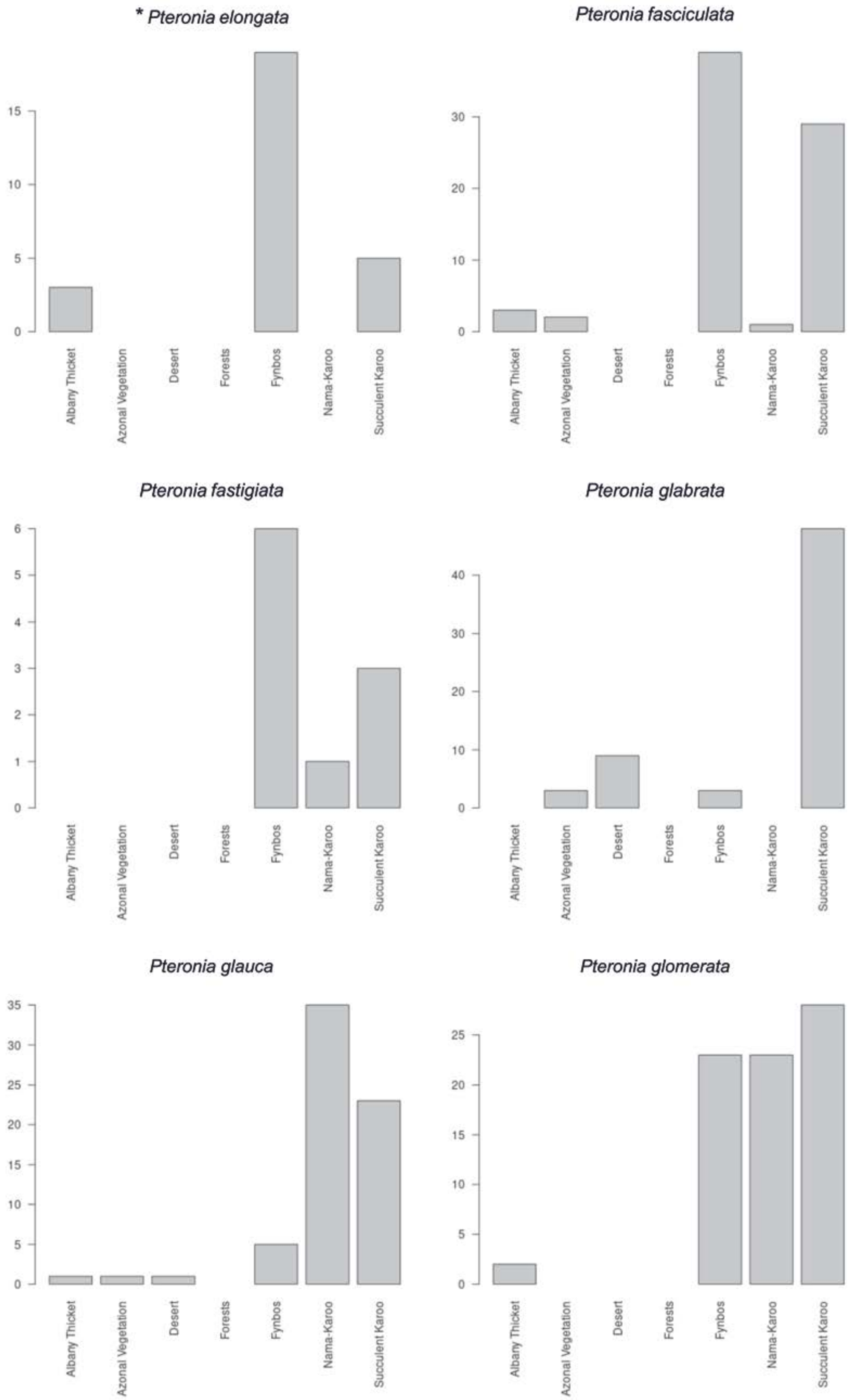

Supplementary Figure 7 continued

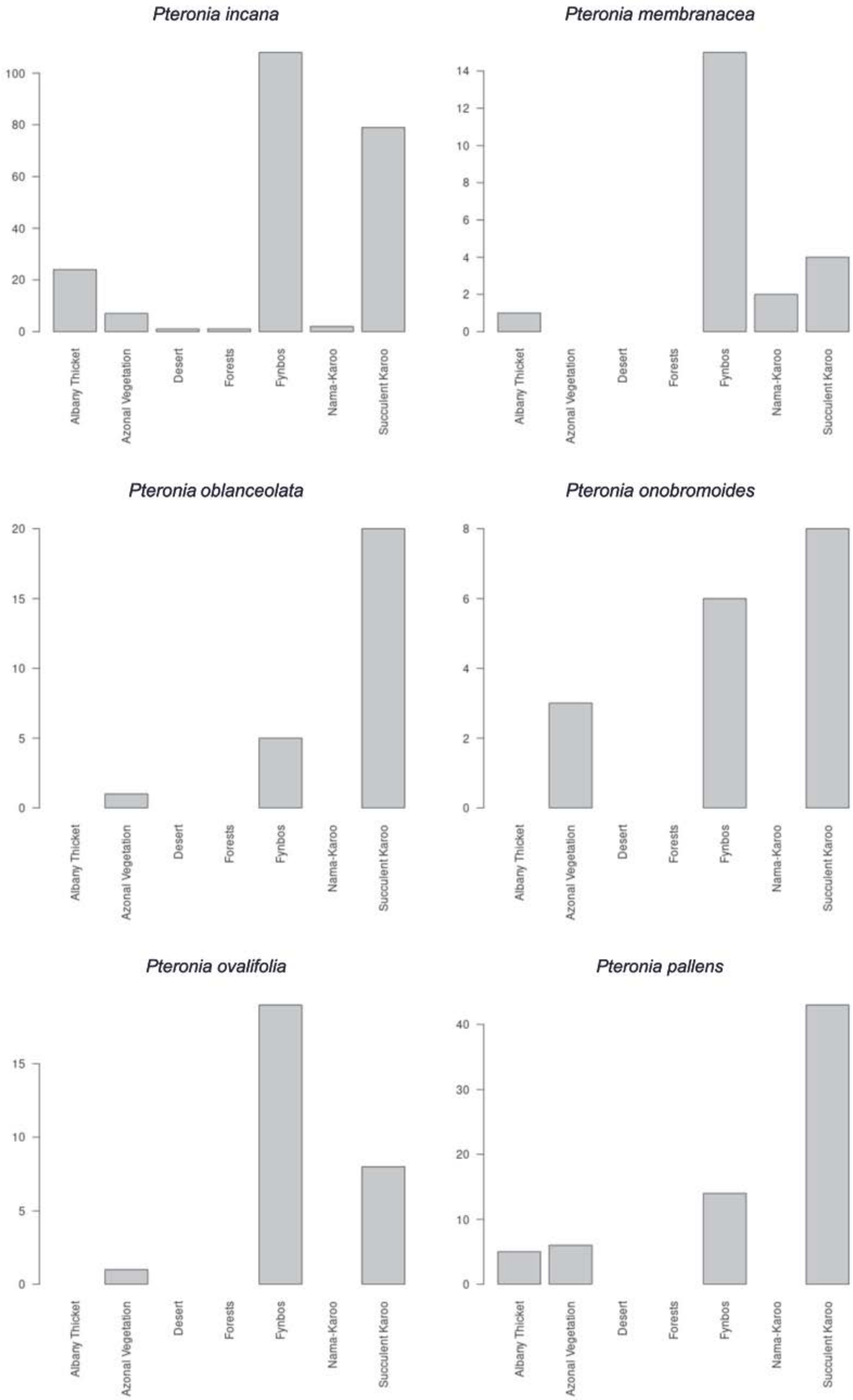

Supplementary Figure 7 continued

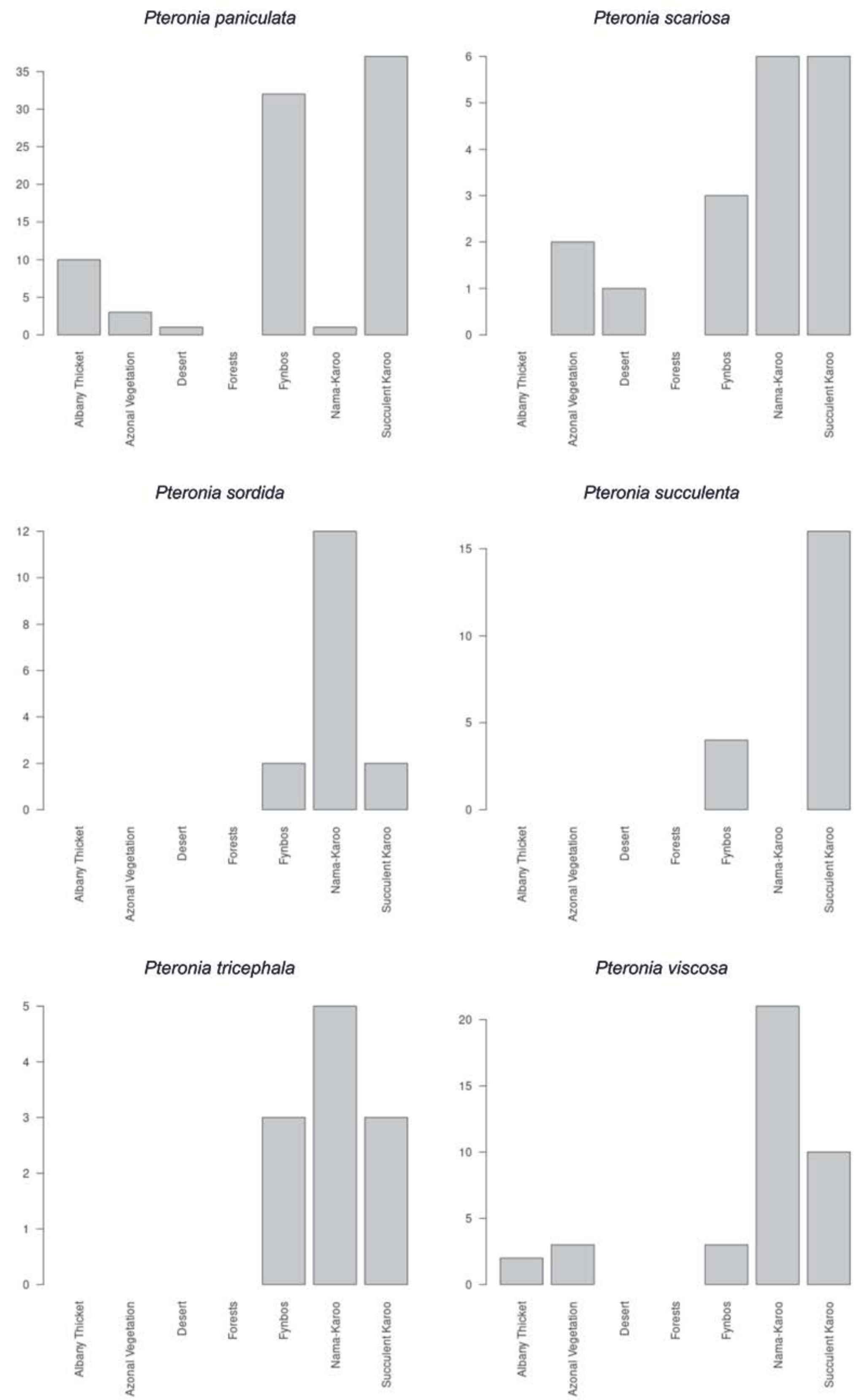

Supplementary Figure 7 continued
